# Antitumor Profile of Carbon-Bridged Steroids (CBS) and Triterpenoids

**DOI:** 10.3390/md19060324

**Published:** 2021-06-03

**Authors:** Valery M. Dembitsky, Tatyana A. Gloriozova, Vladimir V. Poroikov

**Affiliations:** 1 Centre for Applied Research, Innovation and Entrepreneurship, Lethbridge College, 3000 College Drive South, Lethbridge, AB T1K 1L6, Canada; 2 Institute of Biomedical Chemistry, Bldg. 8, 10 Pogodinskaya Str., 119121 Moscow, Russia; tatyana.gloriozova@ibmc.msk.ru (T.A.G.); vladimir.poroikov@ibmc.msk.ru (V.V.P.)

**Keywords:** carbon-bridged steroids, cyclopropane, cyclobutane, cyclopentane, cyclohexane, triterpenoids, pharmacology, antitumor, marine invertebrates, green and red algae, fungi

## Abstract

This review focuses on the rare group of carbon-bridged steroids (CBS) and triterpenoids found in various natural sources such as green, yellow-green, and red algae, marine sponges, soft corals, ascidians, starfish, and other marine invertebrates. In addition, this group of rare lipids is found in amoebas, fungi, fungal endophytes, and plants. For convenience, the presented CBS and triterpenoids are divided into four groups, which include: (a) CBS and triterpenoids containing a cyclopropane group; (b) CBS and triterpenoids with cyclopropane ring in the side chain; (c) CBS and triterpenoids containing a cyclobutane group; (d) CBS and triterpenoids containing cyclopentane, cyclohexane or cycloheptane moieties. For the comparative characterization of the antitumor profile, we have added several semi- and synthetic CBS and triterpenoids, with various additional rings, to identify possible promising sources for pharmacologists and the pharmaceutical industry. About 300 CBS and triterpenoids are presented in this review, which demonstrate a wide range of biological activities, but the most pronounced antitumor profile. The review summarizes biological activities both determined experimentally and estimated using the well-known PASS software. According to the data obtained, two-thirds of CBS and triterpenoids show moderate activity levels with a confidence level of 70 to 90%; however, one third of these lipids demonstrate strong antitumor activity with a confidence level exceeding 90%. Several CBS and triterpenoids, from different lipid groups, demonstrate selective action on different types of tumor cells such as renal cancer, sarcoma, pancreatic cancer, prostate cancer, lymphocytic leukemia, myeloid leukemia, liver cancer, and genitourinary cancer with varying degrees of confidence. In addition, the review presents graphical images of the antitumor profile of both individual CBS and triterpenoids groups and individual compounds.

## 1. Introduction

In both natural and synthetic steroids, when an additional ring is formed within the steroid skeleton, through a direct bond between any two carbon atoms (or more) of the steroid ring system or an attached side chain, such steroids (or triterpenoids) are called carbon-bridged steroids [1,2]. Analyzing the literature data from 1920, we concluded that the first mention of cyclopropane-containing hormones appeared in the mid-1930s of the twentieth century [2,3,4]. Steroids containing a cyclopropane ring in the side chain, such as gorgosterol, were first isolated from marine organisms in the early 1940s [4,5,6], and other 22,23-cyclopropyl sterols, such as dimethyl-gorgosterol, acanthasterol, demethylacanthasterol, acanthastanol, and 9,11-secogorgosterol, all of which have 22*R*, 23*R* and 24*R* configurations, have been isolated from marine sources [7,8,9,10,11,12]. Natural triterpenes containing a cyclopropane ring, and called cycloartanes, were first found in the early 1950s [13,14,15].

Natural carbon-bridged steroids predominantly contain an additional cyclopropane ring, and to a lesser extent cyclobutane, cyclopentane, cyclohexane or cycloheptane, although synthetic CBS can contain a wide variety of additional rings. It was found that all these groups of CBS exhibit a wide range of biological activities [16,17,18,19,20,21].

Over the past 30–40 years, scientists have made great efforts to search for antitumor agents, among both natural and synthetic compounds, for use in practical and experimental medicine [22,23,24,25,26,27,28,29,30,31,32,33,34,35,36,37,38,39,40,41,42,43,44,45,46]. In our opinion, natural and synthetic carbon-bridged steroids or similar triterpenoids can be excellent anticancer agents, as they exhibit a wide range of biological activities and, predominantly, antitumor activity.

Our review focuses on this topic, and we consider about 300 natural, semi-, and synthetic carbon-bridged steroids and similar triterpenoids, many of which show pronounced antitumor activity.

## 2. Cyclopropane Containing Steroids and Triterpenoids

**Figure 1:**
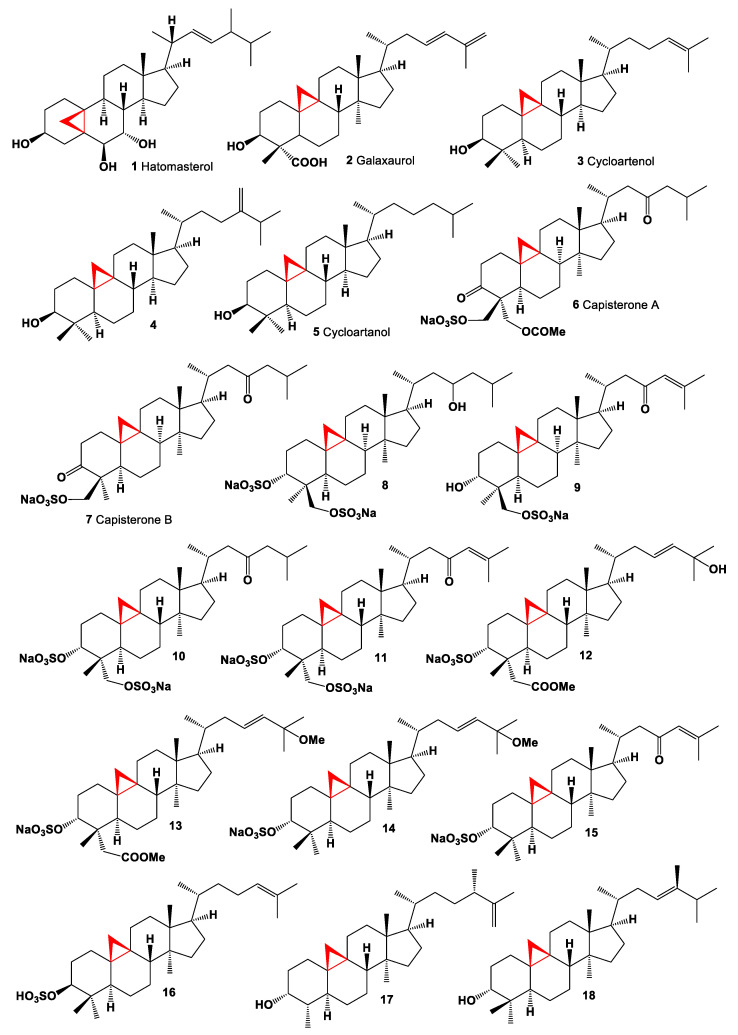
Bioactive steroids containing an additional 3-membered ring in molecule.

A unique steroid containing a 5,19-cycloergostane skeleton, (3β,5β,6β,7α,22*E*,24ξ)-5,19-cycloergost-22-ene-3,6,7-triol, named hatomasterol (**1**) was found in the extracts of the Okinawan sponge *Stylissa* sp., and an isolated compound demonstrated cytotoxicity against HeLa cells in vitro [47]. Chemical structures **1**–**18** are shown in Figure 1, and their biological activity is shown in Table 1.

Cycloartane derivatives are widely distributed in terrestrial plants, but only a few were obtained from the seaweeds and marine invertebrates. Thus, cycloartane triterpene 3-hydroxy-cycloarta-23,25-dien-28-oic acid (**2**) was found in the red alga *Galaxaura* sp. [48]. Cycloartenol (**3**), 24-methylene cycloartenol (**4**), and cycloartanol (**5**) have been detected in brown alga *Fucus spiralis* and *F. krishnae* (Phaeophyceae) [49,50], in the marine green algae *Enteromorpha intestinalis* and *Ulva lactuca* [51], in a freshwater species of single-celled alga *Euglena gracilis* [52], in a yellow-green unicellular freshwater alga *Monodus subterraneus* [53], and in the subarctic moss *Dicranum elongatum* [54]. Cycloartenol (**3**) was also found in a single-cell green alga *Chlamydomonas reinhardtii* [55], a single-celled green algae *Chlorella ellipsoidea* [56], and cycloartenol is found in a ubiquitous green alga *Prototheca wickerhamiiin* [57], in the marine alga *Aurantiochytrium* sp. [58], and in the red seaweed *Laurencia dendroidea* [59].

Interestingly, cycloartenol (**3**) is the sterol precursor in photosynthetic organisms such as amoebae *Naegleria lovaniensis*, *N. gruberi* and the soil amoeba *Acanthamoeba polyphaga* using [l-^14^C] acetate in the biosynthesis of all steroids in the genus Amoeba [60,61]. In addition to cycloartenol, 24-methylene cycloartenol (**4**), cycloartanol (**5**), and 31-norcycloartenol (**34**) were also identified using NMR spectra in *Naegleria lovaniensis, N. gruberi* (Milankovic 2017) [62], and *Acanthamoeba polyphaga* [63], and cycloartenol was found in the amoeba *Dictyostelium discoideum* [57].

The crude aqueous and EtOAc extracts of tropical Atlantic green alga *Penicillus capitatus* (Bryopsidales) showed potent inhibition of the ubiquitous marine fungal pathogen *Lindra thallasiae*. The authors studied the lipid composition and found two sulphate esters named capisterones A (**6**) and B (**7**) [64]. The MeOH extract of the green alga *Tuemoya* sp. showed inhibitory activity against Herpes Zoster protease, and the extract yielded two steroids, cycloartane-3,28-disulfate-23-ol (**8**) and cycloart-24-en-23-one-28-sulfate-3-ol (**9**). Both compounds demonstrated activity against both VZV and CMV protease in the 4–7 μM range [65]. Three cycloartenol sulfates (**8**, **10**, and **11**) that inhibit protein tyrosine kinase pp60v-src were isolated from a tropical deep-water siphonaceous green alga *Tydemania expeditions* [66].

Four steroids, 3β-methyl-25-dihydroxycycloart-23-en-29-oate 3-sulfate (**12**), 3β-methyl-hydroxy-25-methoxycycloart-23-en-29-oate 3-sulfate (**13**), 3β-hydroxy-25-methoxycycloart-23-ene 3-sulfate (**14**) and (3β-hydroxycycloart-24-en-23-one 3-sulfate (**15**) were isolated from Vietnamese red alga *Tricleocarpa fragilis*. All isolated steroids showed potent inhibitory activity against yeast α-glucosidase with IC_50_ values of 16.6, 36.3, 30.2 and 6.5 µM, respectively [67]. The Far Eastern sea cucumber *Eupentacta fraudatrix* (Class Holothuroidea) are sedentary and feed on plankton, algae, and organic debris extracted from bottom silt and sand that is passed through the alimentary canal. Sulfated cycloartane (**16**), which was found in sea cucumber extract, appears to be a metabolite of algae origin [68].

**Figure 2:**
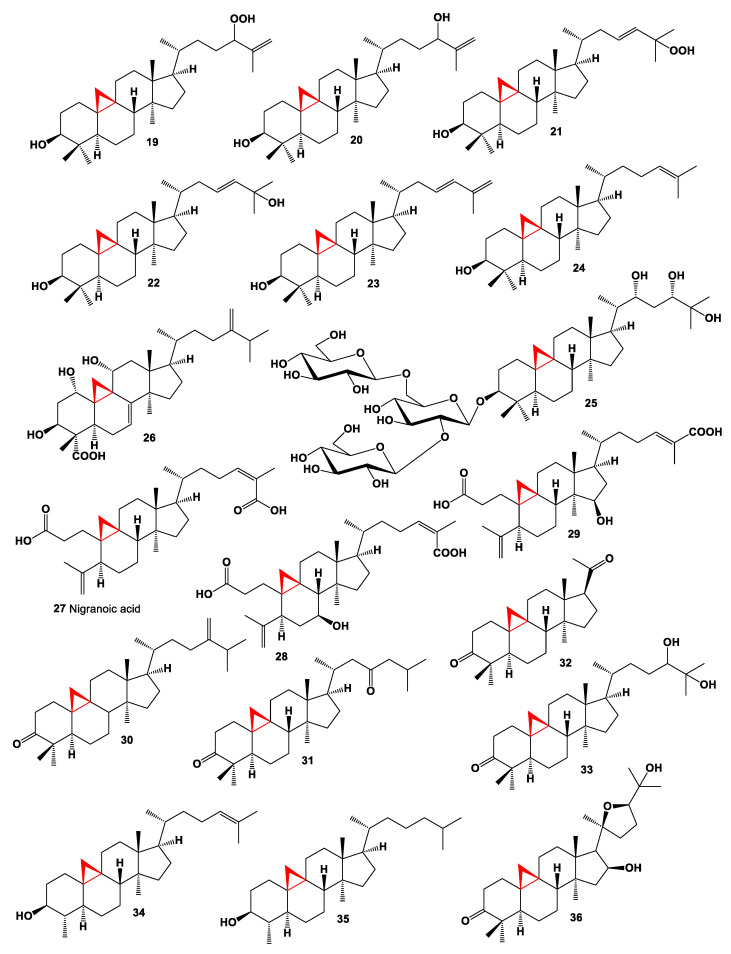
Bioactive steroids containing an additional 3-membered ring in the steroid molecule.

Two cycloartane-type triterpenoids, 3-epicyclomusalenol (**17**), and cyclosadol (**18**) were isolated from brown algae *Kjellmaniella crassifolia*. Both compounds have been reported to have moderate chemo preventive effects [69,70]. Six cycloartanes, 24-hydroperoxycycloart-25-en-3β-ol (**19**, chemical structures **19**–**36** are shown in Figure 2, and their biological activity is shown in Table 2), cycloart-25-en-3β24-diol (**20**), 25-hydroperoxycycloart-23-en-3β-ol (**21**), cycloart-23-en-3β,25-diol (**22**), cycloart-23,25-dien-3β-ol (**23**), and cycloart-24-en-3β-ol (**24**) were isolated from ethanol extract of marine green alga *Cladophora fascicularis* [71]. The small, floating plant *Spirodela punctata* (or *Landoltia punctata*, also known as dotted duckmeat) is widespread in the Hawaiian Islands, Southern and Eastern United States, and synthesized cycloartane glycoside (**25**). The biological activity of this glycoside has not been studied [72].

The uncommon 24-homo-30-nor-cycloartane (**26**), produced by the endophytic fungus *Mycoleptodiscus indicus* FT1137, which was isolated from the Hawaiian *Stenocereus* sp. (family Cactaceae). Obtained compound demonstrated cytotoxic activity against human ovarian cancer cell line A2780 [73]. An endophytic fungus *Trichoderma harzianum* which isolated from *Kadsura angustifolia* produce 3,4-secocycloarta-4(28),24-(Z)-diene-3,26-dioic acid named nigranoic acid (**27**) and its highly oxygenated derivatives [74], and another endophytic fungus *Umbelopsis dimorpha* transformed the triterpene nigranoic acid into its derivatives (**28**) and (**29**) [75]. A steroid called cycloeucalenone (**30**) was isolated from an unidentified fungus collected from New Jersey [76]. Akihisa and co-workers reported that the fungus *Glomerella fusarioides* transformed cycloartenol (**4**) to cycloartane-3,24-dione (**31**), rare 4α,4β,14α-trimethyl-9β,19-cyclopregnane-3,20-dione (**32**) and 24,25-dihydroxycycloartan-3-one (**33**) [77].

31-Norcycloartenol (**34**) and cycloartanol (**5**) are found in a fern oil from the family Polypodiaceae, *Polypodium vulgare* [78], and 29-nor-cycloartanol (**35**) and cycloartanol (**5**) was detected in a flowering plant in the spurge family Euphorbiaceae, *Euphorbia balsamifera* [79].

The *Parthenium argentatum* (commonly known as guayule) extract contained a cytotoxic steroid named argentatin A (**36**), which showed a cytotoxic effect against the human colon cancer cell lines (HCT15, HCT116, and SW620) and normal epidermal keratinocytes cell line [80].

**Figure 3:**
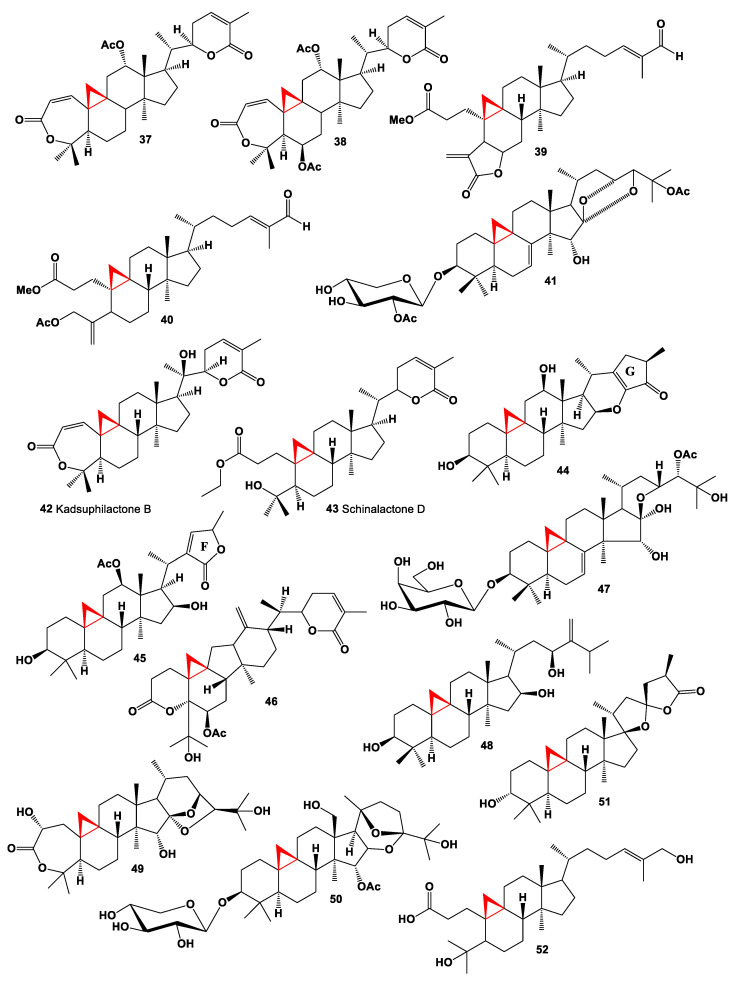
Bioactive steroids containing an additional 3-membered ring in the steroid molecule.

The triterpenoids named xuetonglactones E (**37**, chemical structures **37**–**52** are shown in Figure 3, and their biological activity is shown in Table 3) and F (**38**) were isolated from the stems of an evergreen climbing shrub *Kadsura heteroclita*. Both compounds showed potent cytotoxic activities against human cervical cancer cell lines (HeLa) and human gastric cancer cells (BGC 823) [81]. The rare ring-A seco-cycloartane carbon skeleton, coronalolide methyl ester (**39**), and methyl coronalolate acetate (**40**) were isolated from the leaves and stems of *Gardenia coronaria*. Both compounds showed broad cytotoxic activity when evaluated against a panel of human cancer cell lines [82]. Cytotoxic cycloartane triterpenoid, 25-*O*-acetyl-7,8-didehydrocimigenol-3-*O*-β-d-(2-acetyl)-xylopyranoside (**41**) was found from *Cimicifuga foetida* [83]. This compound demonstrated antitumor activity against cancerous MCF-7, HepG2/ADM, HepG2 and HELA cell lines. A medicinal plant *Schisandra chinensis* contains two triterpenoids, kadsuphilactone B (**42**), and schinalactone D (**43**), which showed anti-HIV-1 activity and antitumor effects [84].

Cycloartane derivatives, cimyunnin A (**44**) with an unusual fused cyclopentenone ring G, together with cimyunnin D (**45**), possessing a highly rearranged c-lactone ring F, were found in the fruit of *Cimicifuga yunnanensis* and their structures were determined using physical-chemical methods [85]. 3,4-Seco-cycloartane triterpenoid which had rearranged 5/6 consecutive carbocycle rings C/D, named ananosins A (**46**), was isolated from the stems of *Kadsura ananosma* [86].

Cycloartenol triterpene saponin, 7,8-didehydro-24*S*-*O*-acetylhydroshengmanol-3-*O*-β-d-galactopyranoside named shengmaxinside C (**47**) has been isolated from the ethyl acetate soluble fraction of an ethanol extract of *Cimicifuga simplex* roots [87]. A 24-methylene-cycloartane-3β,16β,23β-triol, named longitriol (**48**) was isolated from ethanolic extract of the leaves of *Polyalthia longifolia* var. *pendula*, and shown cytotoxic effects against four human cancer cell lines and found to be most active against cervical carcinoma cell lines [88].

The aerial parts of *Cimicifuga heracleifolia* contained a 9,19-cycloartane-type triterpene, cimiheraclein A (**49**) and showed weak activity against human tumor cell lines (HL-60, SMMC-7721, A-549, MCF-7, and SW-480) [89]. The rhizomes of *Beesia calthifolia* resulted in the isolation of cycloartane derivative (**50**) [90], and *Abies faxoniana* is the source of cycloartane derivative (**51**) with spiro-side chain [91]. The 3,4-seco-cycloartane, macrocoussaric acid F (**52**) has been isolated from *Coussarea macrophylla* [92].

**Figure 4:**
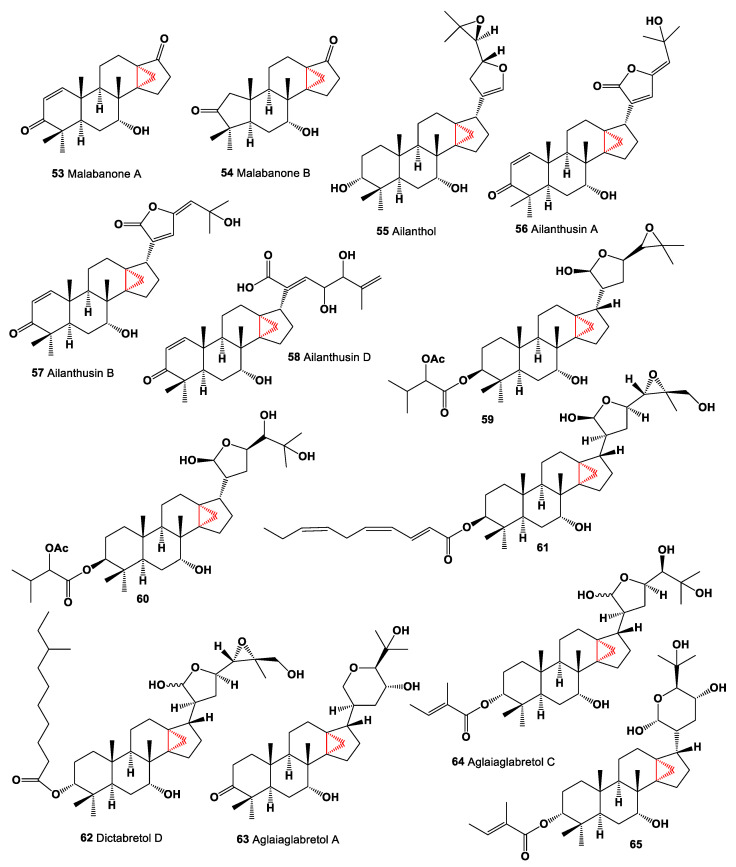
Bioactive steroids containing an additional 3-membered ring in the steroid molecule.

Unique steroids, 4,4,8β-Trimethyl-7α-hydroxy-13α, 14α-methano-18-nor-5α-androsta-1-ene-3,17-dione, named malabanone A (**53**) and 3,3,8β-trimethyl-7α-hydroxy-13α,14α-methano-A (4),18-dinor-5α-androstane-2,17-dione named malabanone B (**54**), which incorporate a unique tricyclo [4.3.1.01,6] decane unit in the structures, were isolated from the stem bark of *Ailanthus malabarica*. The authors suggest that both steroids are biosynthesized from ailanthol (**55**, (23*R*,24*S*)-4,4,8β-Trimethyl-13α,14α-methano-21,23:24,25-diepoxy-18-nor-5α-cholesta-20-ene-3α,7α-diol), which was also isolated from this plant [93]. Chemical structures **53**–**65** are shown in Figure 4, and their biological activity is shown in Table 4.

Several steroids with an incorporated cyclopropane unit at positions 14 and 18 named ailanthusins A (**56**), B (**57**) and D (**58**) have been found and isolated from the CH_2_Cl_2_ extracts of Thailand rainforest tree *Ailanthus triphysa* [94]. The dichloromethane extract of the air-dried leaves of *Dysoxylum mollissimum* afforded two glabretal-type triterpenoids (**59** and **60**) [95]. Cytotoxic glabretal triterpene, pancastatin B (**61**) was detected in the immature fruits of *Poncirus trifoliata*. This compound exhibited selective cytotoxicity against PANC-1 pancreatic cancer cells under low-glucose stress conditions [96]. Another glabretal-type triterpenoid named dictabretol D (**62**) was isolated by activity-guided fractionation from the root bark of *Dictamnus dasycarpus* (Rutaceae). This triterpenoid demonstrated inhibition of proliferation of activated T cells [97]. A CHCl_3_-MeOH extract of the bark of *Aglaia crassinervia* collected in Indonesia led to the isolation of aglaiaglabretols A (**63**) and C (**64**) [98], and derivative (**65**) of aglaiaglabretols A was found in the stems of *Spathelia excelsa* (Rutaceae) [99], and it exhibited larvicidal properties with LC_50_ of 4.8 µg/mL against yellow fever mosquito, *Aedes aegypti*.

**Figure 5:**
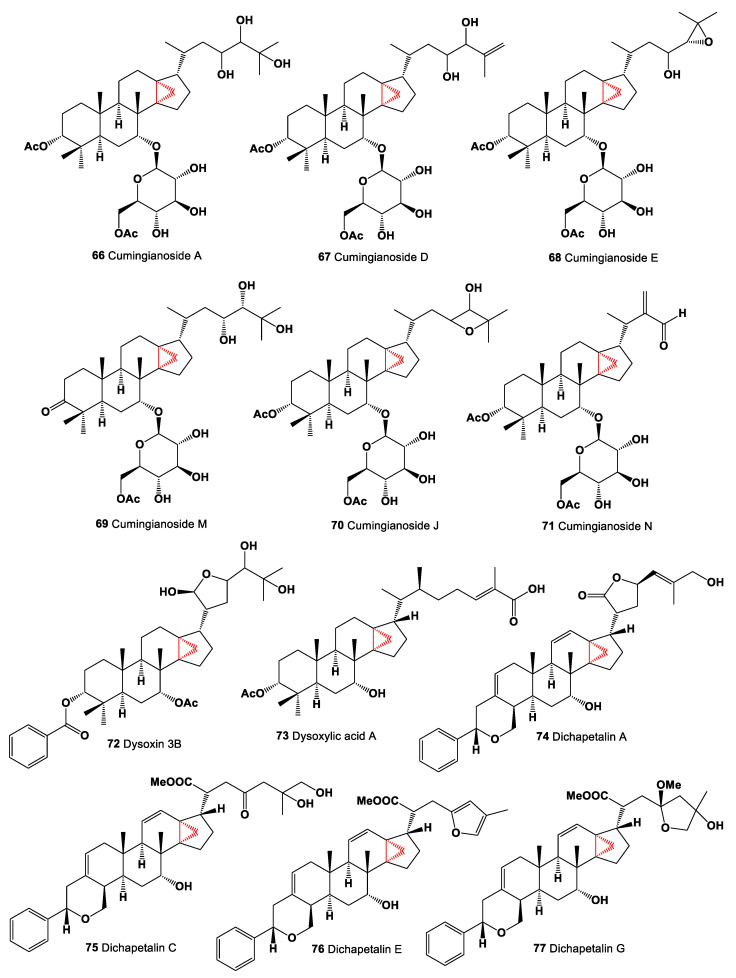
Bioactive steroids containing an additional 3-membered ring in the steroid molecule.

Series of antitumor triterpene glucosides, named cumingianosides A (**66**, chemical structures **66**–**77** are shown in Figure 5, and their biological activity is shown in Table 5), D (**67**), E (**68**), M (**69**), J (**70**) and N (**71**) containing a 14,18-cycloapotirucallane-type skeleton were isolated from a cytotoxic fraction of the leaves of *Dysoxylum cumingianum*. The cytotoxic activity of cumingianosides showed that cumingianoside M (**69**) exhibited significant (<4 μM) cytotoxicity, especially against leukemia and melanoma cell lines [100,101].

**Figure 6:**
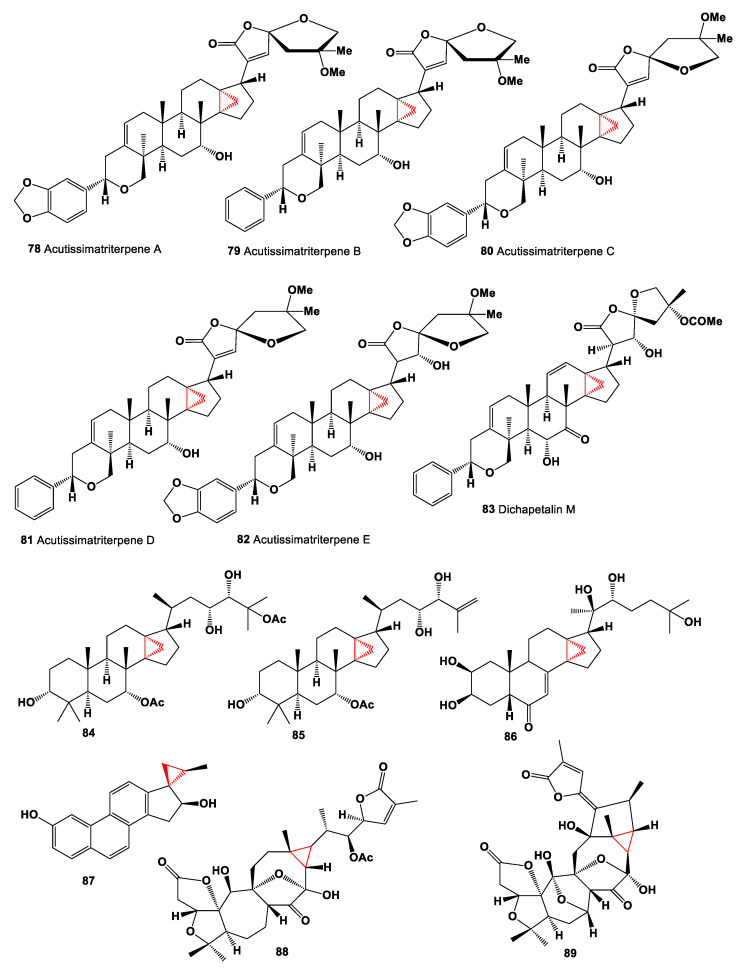
Bioactive cyclopropane-containing steroids and meroterpenoids.

A hexane extract of the wood of *Dysoxylum muelleri* has a yielded triterpenoid called dysoxin 3b (**72**), and dysoxylic acid A (**73**) was isolated from the hexane extract of the wood and bark of *Dysoxylum pettigrewianum* [102,103]. Dichapetalins are a small group of triterpenoids found primarily in the Dichapetalaceae and Euphorbiaceae. Thus, bioactive dichapetalins A (**74**), C (**75**), E (**76**), and G (**77**) were found in extracts of the roots of *Dichapetalum madagascariense*, and dichapetalin A (**74**) showed a strong and selective cytotoxic activity [104,105]. The aerial parts of *Phyllanthus acutissima* contained in CH_2_Cl_2_ extracts of several dichapetalin-type triterpenoids, acutissimatriterpenes A (**78**, chemical structures **78**–**89** are shown in Figure 6, and their biological activity is shown in Table 6), B (**79**), C (**80**), D (**81**), and E (**82**). The obtained compounds were demonstrated cytotoxic and anti-HIV-1 activities [106]. The 90% MeOH-soluble fraction of the leaves of *Dysoxylum cumingianum* led to the isolation of triterpenoids (**84** and **85**), which showed significant enhanced cytotoxicity in the presence of colchicine, indicating that they might have some MDR-reversal effect [107].

Natural ecdysteroids are found in marine invertebrates, insects, or plants, and they provide a remarkable resource of insect hormone analogues that influence insect development and metamorphosis and thus play a significant role in the chemical interactions between some marine invertebrates and insects [108]. Rare 14-deoxy-14,18-cyclo-20-hydroxyecdysone (**86**) was obtained by photochemical transformation of 20-hydroxyecdysone [109].

Cinanthrenol A (**87**), an estrogenic aromatic steroid containing a phenanthrene and a spiro[2,4]heptane systems has been isolated from a marine sponge *Cinachyrella* sp. [110].

Preschisanartanin (**88**) possessing a complex nortriterpenoid skeleton, was isolated from *Schisandra chinensis*, and demonstrated anti-HIV-1 activity with an EC_50_ value of 13.8 μg/mL [111,112,113], and lancolide A (**89**), highly oxygenated Schisandra nortriterpenoid was detected in the *Schisandra lancifolia*. This compound exhibited specific antiplatelet aggregation induced by platelet-activating factor [114].

**Figure 7:**
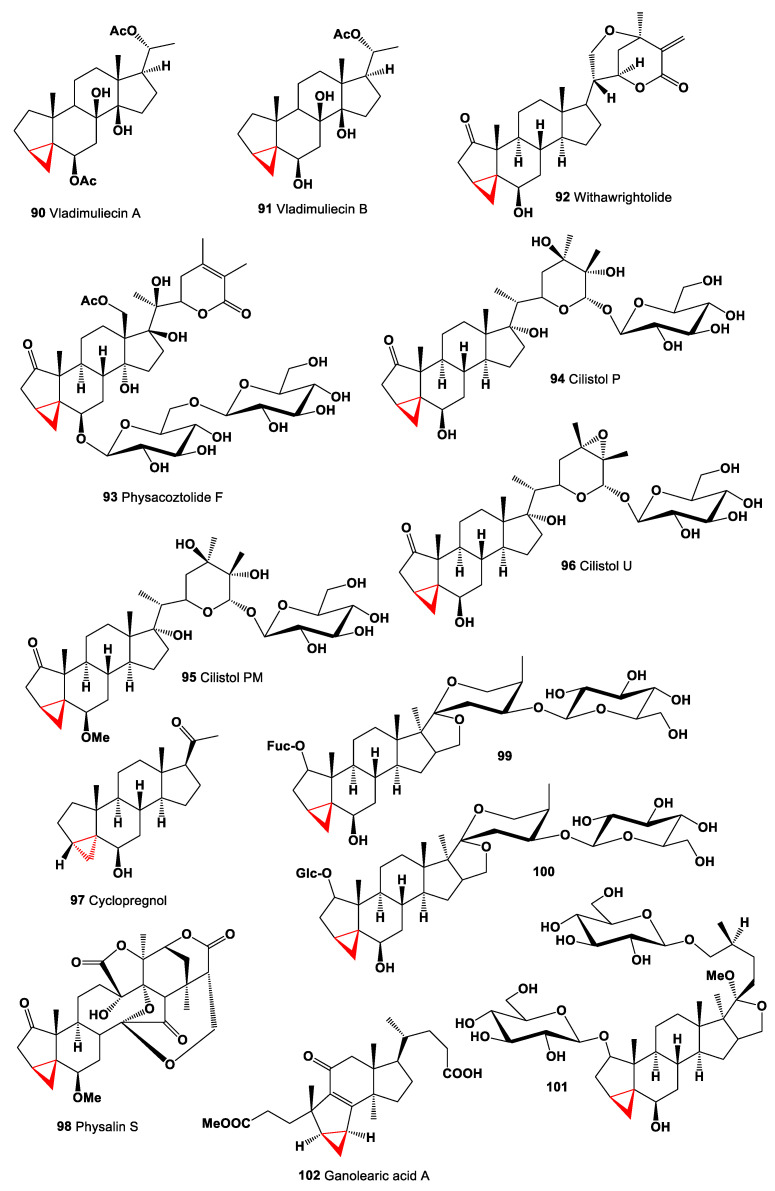
Bioactive cyclopropane-containing steroids.

A pentacyclic 3α,5α-cyclopregnane-type framework steroids represent a small group of natural lipids related to carbon-bridged steroids. These steroids have been found in both marine invertebrates and some terrestrial species. Two cytotoxic steroids, vladimuliecins A (**90**) and B (**91**), were isolated from the rhizome of *Vladimiria muliensis*. Both steroids demonstrated the cytotoxicity against cancer cell lines, including human leukemia cell (HL-60), human hepatoma cell (SMMC-7721), and human cervical carcinoma cell (HeLa) lines [115]. Chemical structures **90**–**102** are shown in Figure 7, and their biological activity is shown in Table 7.

An unusual steroid, named withawrightolide (**92**), was detected and isolated from the aerial parts of *Datura wrightii* (family Solanaceae). Isolated steroid showed antiproliferative activities against human glioblastoma (U251 and U87), head and neck squamous cell carcinoma (MDA-1986), and normal fetal lung fibroblast (MRC-5) cancer cell lines [116]. Glycoside, 6β-*O*-[β-d-glucopyranosyl-(1->6)-β-d-glucopyranosyl]-(20*S*,22*R*)-14α,17β,20-trihydroxy-18-acetoxy-3α,5α-cyclo-1-oxowitha-24-enolide, named physacoztolide F (**93**), was found in the CH_2_Cl_2_/MeOH extract of the aerial parts of *Physalis coztomatl* (family Solanaceae) [117]. Withanolide-type steroids named cilistols P (**94**), PM (**95**) and U (**96**) were isolated from the leaves of *Solanum cilistum* [118]. Psychotropic agent, 6β-hydroxy-3:5-cyclopregnan-20-one (**97**) also known as cyclopregnol was developed in the 1950s [119].

The physalins are a group of 13,14-seco-16,24-cycloergostane triterpenoids, which are produced by the Physalis species [120], and physalin S (**98**), isolated from the *Physalis alkekengi* var. *francheti*, had a 6β-hydroxy-3,5-cyclo arrangement, a common acid rearrangement product of 3-hydroxy-D5 steroids [121]. Steroidal compounds contained in *Dracaena surculosa* (family Agavaceae) led to the isolation of two 3,5-cyclospirostanol saponins (**99** and **100**) and 3,5-cyclofurostanol saponin (**101**) [122].

Ganolearic acid A (**102**), a 3,4-seco-hexanortriterpenoid featuring, a rare 3/5/6/5 tetracyclic system with anti-inflammatory activity, was obtained in trace amounts from *Ganoderma cochlear* [123].

## 3. Sterols and Triterpenoids with Cyclopropane Ring in the Side Chain

**Figure 8:**
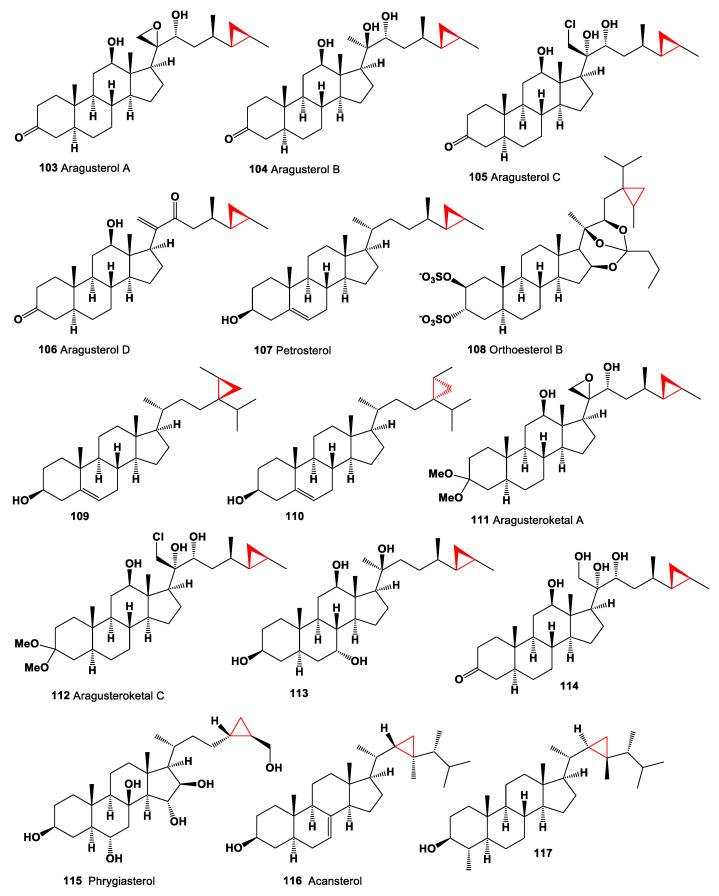
Bioactive sterols and triterpenoids with cyclopropane ring in the side chain.

A cytotoxic steroid, aragusterol A (**103**, chemical structures **103**–**117** are shown in Figure 8, and their biological activity is shown in Table 8), which possessing potent antitumor activity, was isolated from the Okinawan sponge of the genus Xestospongia. The compound strongly inhibited the cell proliferation of KB, HeLaS3, P388, and LoVo cells in vitro, and showed potent in vivo antitumor activity toward P388 in mice and L1210 in mice [124]. Additionally, 26,27-cyclosterols aragusterols B (**104**), C (**105**), and D (**106**) have been identified and isolated from the Okinawan marine sponge of the genus *Xestospongia* [124,125]. Steroids, aragusterol A (**103**), petrosterol (**107**), orthoesterol B (**108**), and other cyclopropyl containing steroids (**109** and **110**) were isolated from the marine sponge *Petrosia weinbergi* [126]. In additional, 24,28-Methylenestigmast-5-en-3-ol (**109**) was detected in extracts of the marine chrysophyte alga, and pelagophtic alga *Pulvinaria* sp. [127,128].

Marine steroids having dimethylketal structure and named aragusteroketals A (**111**) and C (**112**) with cytotoxic activity have been isolated from an Okinawan marine sponge of *Xestospongia* sp. [125]. Many steroids have been found in the marine sponge *Petrosia (Strongylophora)* sp. collected from the Similan Island (Thailand). In addition to the already known steroids aragusterol A (**103**), petrosterol (**107**), and aragusteroketals A (**111**), compounds **113** and **114** were additionally identified [129]. Experimental data showed that aragusterol A (**103**) exhibited weak to moderate cytotoxicity, with the IC_50_ values in the range of 11–103 µM. The most potent was cytotoxic, with the IC_50_ values of 7.1 and 6.1 µM against HepG-2 and HeLa cell lines, respectively, while exhibiting moderate cytotoxicity with the IC_50_ values of 12.8, 37.9, 37.5, and 18.0 µM against the other four cancer cell lines, MOLT-3, A549, HuCCA-1, and MDA-MB-231, respectively. In addition, this compound showed broad-spectrum anti-proliferative activity against a panel of 14 human cancer cell lines (IC_50_ = 0.01–1.6 μM) [130]. A cyclopropane-containing hydroxy sterol, phrygiasterol (**115**), was isolated from starfish *Hippasteria phrygiana* [131], and an extract of the crown-of-thorns starfish *Acanthaster planci* contained cyclopropane-containing sterol (**116**) [132].

**Figure 9:**
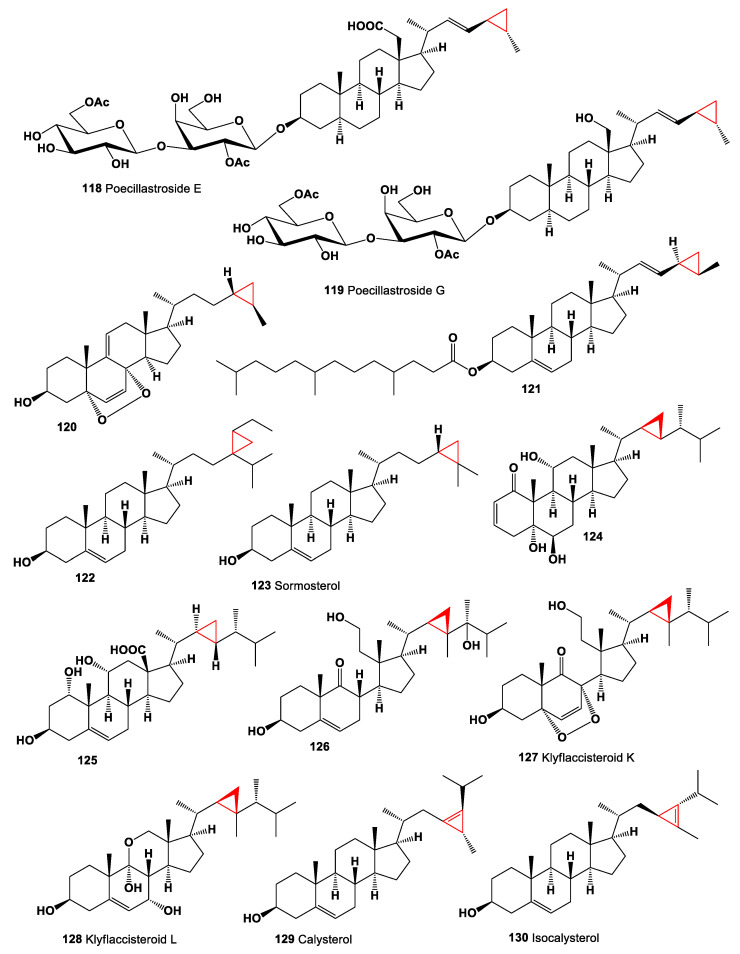
Bioactive sterols and triterpenoids with cyclopropane ring in the side chain.

The steroid, (3β,4α,5α)-4-methylgorgostan-3-ol (**117**), is synthesized by marine algae and invertebrates, and it has been found in dinoflagellates *Peridinium foliaceum* and *Glenodinium foliaceum*, corky sea finger *Briareum asbestinum*, rough leather coral *Sarcophyton glaucum*, and soft coral *Lobophytum* sp. [133,134,135]. Steroidal saponins named poecillastrosides E (**118**) and G (**119**), an oxidized methyl at C-18, into a primary alcohol or a carboxylic acid, have been found in extracts of the Mediterranean deep-sea sponge *Poecillastra compressa*. Poecillastroside E (**118**, chemical structures **118**–**130** are shown in Figure 9, and their biological activity is shown in Table 9), bearing a carboxylic acid at C-18, showed antifungal activity against *Aspergillus fumigatus* [136].

A 5α,8α-epidioxy steroid (**120**) obtained from MeOH extracts of the sponge *Tethya* sp. possessing a cyclopropyl ring at C-24 of the sidechain [137]. Sterol ester, 24,26-cyclo-5α-cholest-(22*E*)-en-3β-4′,8′12′-trimetyltridecanoate (**121**), has been isolated from a deep-water marine sponge, *Xestospongia* sp. [138]. The steroid, (3β,24ξ,28ξ)-29-methyl-24,28-methylenestigmast-5-en-3-ol (**122**) was found in the sponge *Pseudaxinyssa* sp. [139], and another steroid, 25,28-cycloergost-5-en-3-ol, named sormosterol (**123**), was found in the sponge *Lissodendoryx topsenti* [140].

Three steroids, 5,6,11-trihydroxy-33-norgorgost-2-en-1-one (**124**), 1,3,11-trihydroxy-23-norgorgost-5-en-13-oic acid (**125**), and 3,11,24-Trihydroxy-9,11-secogorgost-5-en-9-one (**126**) were isolated from the soft corals *Clavularia viridis, Sinularia dissecta*, and *Pseudopterogorgia* sp., respectively [141,142,143]. Two steroids, klyflaccisteroids K (**127**) and L (**128**), were isolated from a soft coral *Klyxum flaccidum*. Klyflaccisteroid K is a rare 9,11-secosteroid with a 5,8-epidioxy-9-ene functional group, and klyflaccisteroid L has an unusual 11-norsteroid skeleton and is the first example of 11-oxasteroid isolated from natural sources. The compound (**127**) possessed moderate to weak cytotoxicity against multiple cancer cells [144].

A rare steroid named calysterol (**129**), the minor sterol component of the sponge *Calyx niceaensis* and *Petrosia ficiformis*, possessing the unique feature of a cyclopropene ring bridging C23,24 [145,146,147], and isocalysterol (**130**), was detected in the same sponge [148].

**Figure 10:**
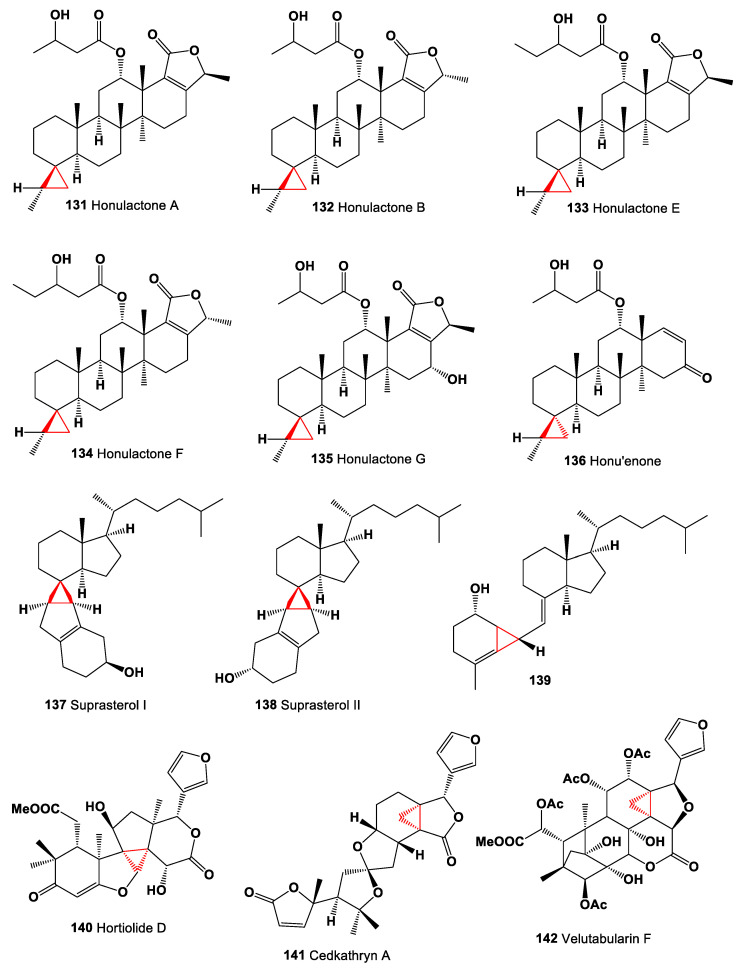
Bioactive cyclopropane-containing steroids and triterpenoids.

The dichloromethane-2-propanol (1:1) extract of the Indonesian marine sponge *Strepsichordaia aliena* yielded 20,24-bishomoscalarane sesterterpenes named honulactones A (**131**), B (**132**), E (**133**), F (**134**), and G (**135**). Honulactones A and B exhibited cytotoxicity against P-388, A-549, HT-29, and MEL-28 (at IC_50_ 1 μg/mL) human tumor cell lines [149], and honu’enone (**136**) [150]. Chemical structures **131**–**142** are shown in Figure 10, and their biological activity is shown in Table 10.

It is known that human skin is responsible for the production of vitamin D. When exposed to ultraviolet radiation, which penetrates the epidermis and photolysis provitamin D3 to previtamin D3, and is photolyzed to 5,6-transvitamin D3 and two cyclopropane-containing derivatives of vitamin D3, suprasterol I (**137**) and suprasterol II (**138**). The resulting photolysis products are used for the treatment and prevention of psoriasis [151]. Mushrooms are a rich source of ergosterol, which is a precursor to vitamin D2. Wild-grown mushrooms have been shown to contain small amounts of vitamin D2. In addition, it is known that the content of vitamin D2 and its derivatives such as suprasterol I and II in cultivated mushrooms increases when exposed to artificial ultraviolet radiation. In addition, vitamin D2 and its derivatives suprasterol I and II have been found in mushrooms *Agaricus bisporus, Pleurotus ostreatus*, and *Lentinula edodes*, as well as several mushroom powders, *Pleurotus eryngii*, and *Agaricus bisporus* [152]. When studying the photosynthesis of vitamin D, using the modelling of non-adiabatic molecular dynamics, another cyclopropane-containing metabolite (**139**) was identified [153].

**Figure 11:**
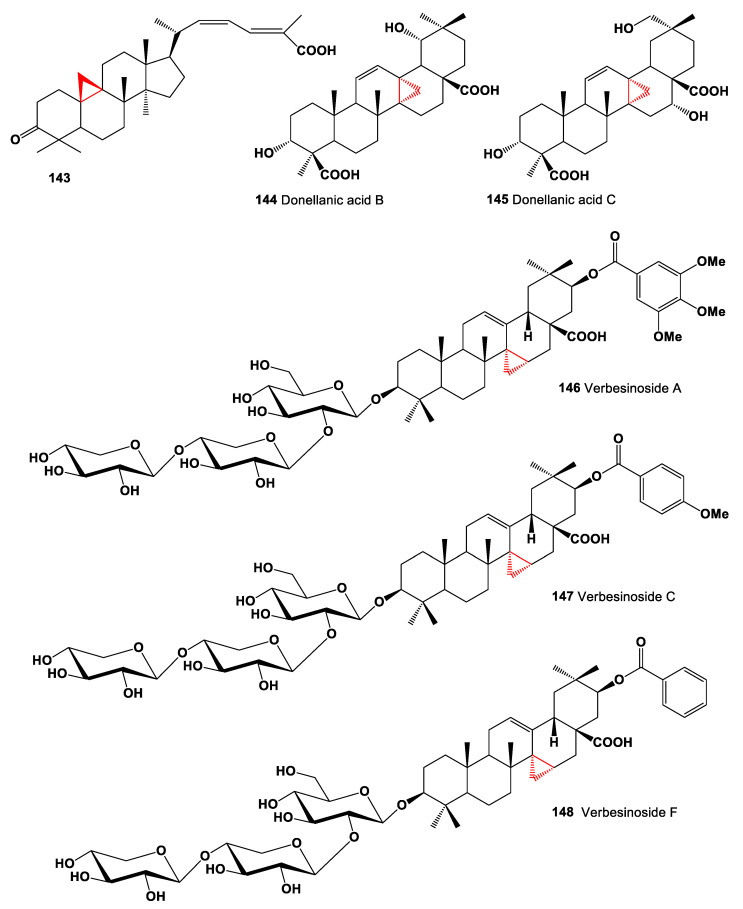
Bioactive steroids containing an additional 3-membered ring in the steroid molecule.

A limonoid named hortiolide D (**140**) was found in CH_2_Cl_2_ and MeOH extracts from the stem of *Hortia oreadica* [154]. The stem bark of *Cedrelopsis gracilis* (Ptaeroxylaceae) has yielded pentanortriterpenoid, cedkathryn A (**141**) [155]. Phragmalin-type limonoid, velutabularin F (**142**) was isolated from the stem bark of *Chukrasia tabularis* var. *velutina* [156]. Rare cytotoxic metabolite, 3-oxo-cycloart-22Z,24E-dien-26-oic acid (**143**) isolated from propolis collected in Myanmar, showed the most potent cytotoxicity against B16-BL6 cell, colon 26-L5, LLC A549, and HeLa HT -1080 cancer cell lines [157]. Chemical structures **143**–**148** are shown in Figure 11, and their biological activity is shown in Table 10.

Two cyclopropanic oleanane triterpenoids named donellanic acid B (**144**) and C (**145**) were obtained from *Donella ubanguiensis*, and its compounds showed cytotoxic and antimicrobial activities [158]. Rare triterpenoid saponins possessing the unique 15,27-cyclooleanane skeleton with different aromatic acyl moieties named verbesinosides A (**146**), C, (**147**) and F (**148**) were isolated from the leaves and flowers of *Verbesina virginica* [159].

**Figure 12:**
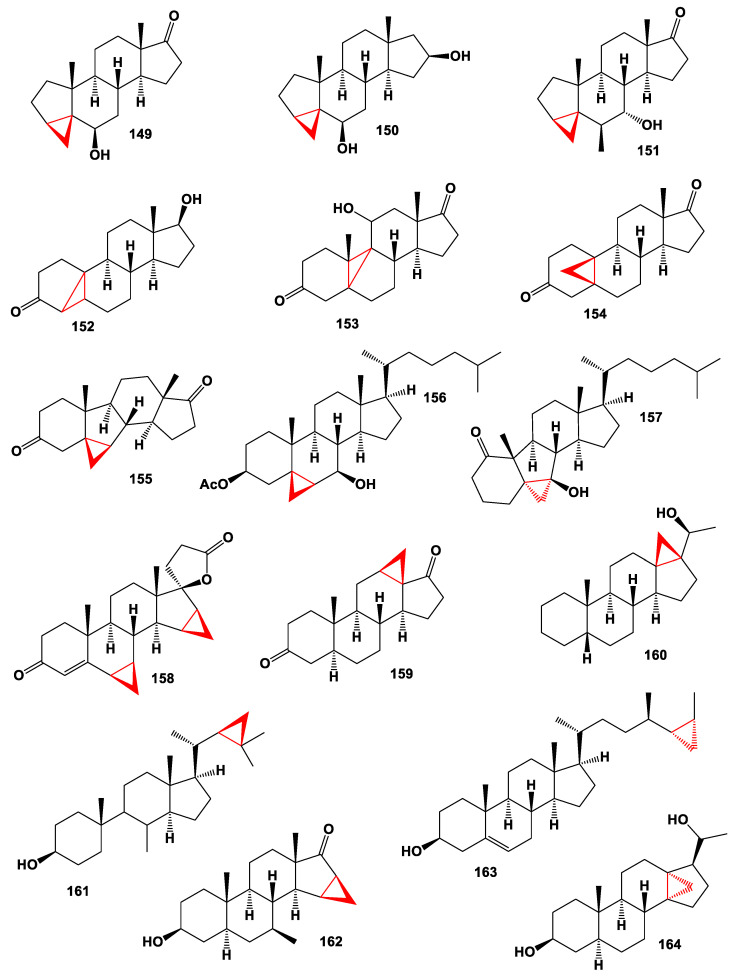
Bioactive synthetic cyclopropane-containing steroids.

It is known that carbon-bridged steroids are a rare group of synthetic lipids that are interesting, both in the beauty of the chemical structure, and show a wide range of biological activities. We have selected several carbon-bridged steroids containing a cyclopropane ring in the molecule that are not found in nature (**149**–**164**, chemical structures **149**–**164** are shown in Figure 12, and their biological activity is shown in Table 11). This is done to compare the biological activities of natural and synthetic steroids [18].

Thus, 6β-hydroxy-3α,5-cyclo-5α-androstan-17-one (**149**), and other analogues (**150**, **151** and **158**) were synthesized as steroidal blood pressure-lowering hormones [160,161]. Cyclosteroids (**152** and **153**), which show an anabolic effect, were synthesized from 19-nor steroids, and would be of great interest for sports medicine as representatives of anabolic steroids [162,163], although other cyclosteroids (**154**–**157**) were synthesized as potential agents with antitumor properties [164,165,166].

A series of cyclopropane containing carbon-bridges steroids (**159**–**164**) have been synthesized in various laboratories, but the biological activity of these lipid molecules has not been determined [160,161,167,168].

## 4. Cyclobutane Containing Steroids and Triterpenoids

The cyclobutane unit is found as a basic structural element in a wide range of naturally occurring compounds in bacteria, fungi, plants, and marine invertebrates [18,19,169,170,171,172,173,174]. The chemistry and biochemistry of cyclobutanes is widely described in the scientific literature and is of great interest to chemists and pharmacologists, since many representatives of this class of compounds demonstrate a wide range of biological activities [18,19,73,175,176,177,178].

**Figure 13:**
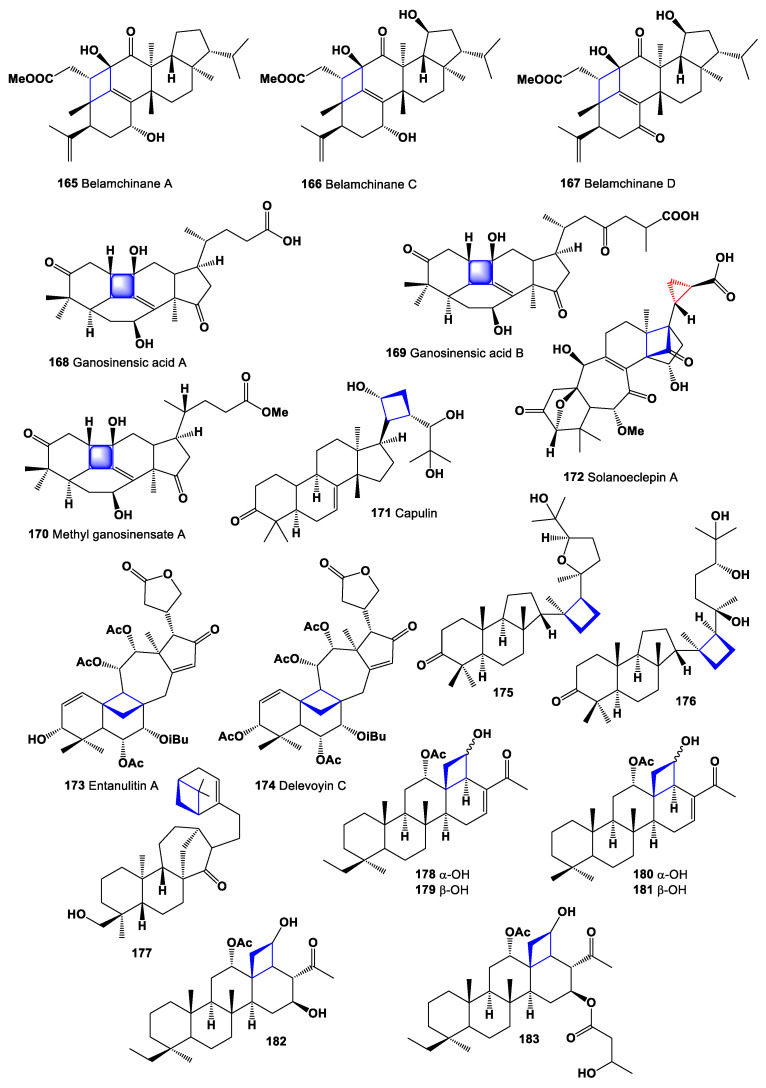
Bioactive cyclobutane-containing steroids and triterpenoids.

Unusual triterpenoids with an unprecedented skeleton named belamchinanes A (**165**), C (**166**), and D (**167**) were isolated from the seeds of *Belamcanda chinensis*. These belamchinanes feature a 4/6/6/6/5 polycyclic system, in which a four-membered carbocyclic ring bridges the C-1 and C-11 positions of a classical triterpenoid framework. Experimental studies showed that **165**-**167** dose-dependently protect age-related renal fibrosis in vitro [179]. Chemical structures **165**–**183** are shown in Figure 13, and their biological activity is shown in Table 12.

Three triterpenoids, with an unusual four-membered ring skeleton, produced by a bond across C-1 to C-11, ganosinensic acid A (**168**), B (**169**), and methyl ganosinensate A (**170**) were isolated from the fruiting body of *Ganoderma sinense* [180]. A protolimonoid named capulin (**171**), containing a four membered ring in its side chain, was isolated from stem barks of *Capuronianthus mahafalensis* (family Meliaceae), endemic to Madagascar [181]. Triterpenoid steroid, named solanoeclepin A (**172**), as a cyst nematode-hatching stimulant, was isolated from potato roots [182].

A rare limonoid named entanutilin A (**173**) was identified from the stem barks of *Entandrophragma utile* collected in Ghana (Africa). This limonoid possessing a cyclobutanyl ring, incorporating C-19 and a cycloheptanyl ring C, including C-30 [183], and the hexane extract of the bark of *Entandrophragma delevoyi* has yielded tetranortriterpenoid, delevoyin C (**174**) with similar skeleton [184].

Unusual two malabaricane type triterpenes, (14S,17S,20S,24R)-25-hydroxy-14,17-cyclo-20,24-epoxy-malabarican-3-one (**175**) и (14S,17S,20S,24R)-20,24,25-trihydroxy-14,17-cyclo-malabarican-3-one (**176**) were isolated from the oleoresin of the wounded trunk, *Ailanthus malabarica* [185]. Unusual triterpenoid bearing a monoterpene unit at C-16 (**177**) has been identified from *Croton limae* (Euphorbiaceae) [186].

Triterpenoids, 12α-acetoxy-13β,18β-cyclobutane-20,24-dimethyl-24-oxoscalar-16-en-25-ol (**178**, α-OH, and **179**, β-OH) was detected in the marine sponge *Phyllospongia papyracea*, collected in Papua New Guinea [187]. Compound (**179**) has also been isolated from the marine Australian sponge *Strepsichordaia lendenfeldi* from Great Barrier Reef [188]. The dichloromethane fraction of the marine sponge *Phyllospongia lamellosa,* collected from the Red Sea, resulted in the isolation and characterization of two scalarane-type compounds, 12α-acetoxy-13β,18β-cyclobutane-24-methyl-24-oxoscalar-16-en-25β-ol (**180**, phyllospongin D) and 12a-acetoxy-13β,18β-cyclobutane-24-methyl-24-oxoscalar-16-en-25α-ol (**181**, phyllospongin E) [189]. The 12α-acetoxy-23,25-cyclo-16β,25-dihydroxy-20,24-dimethyl-24-oxoscalarane (**182**) was isolated from the Neo Guinean sponge *Carteriospongia foliascens* [190,191,192], and similar cyclobutanol-containing metabolite is the related ester, 12α-acetoxy-16β-(3′-hydroxy-butanoyloxy)-13β,18β-cyclobutan-20,24-dimethyl-24-oxosca-laran-25β-ol (**183**) was found in extracts of the Australian sponge *Strepsichordaia lendenfeldi* collected at the Great Barrier Reef [188].

**Figure 14:**
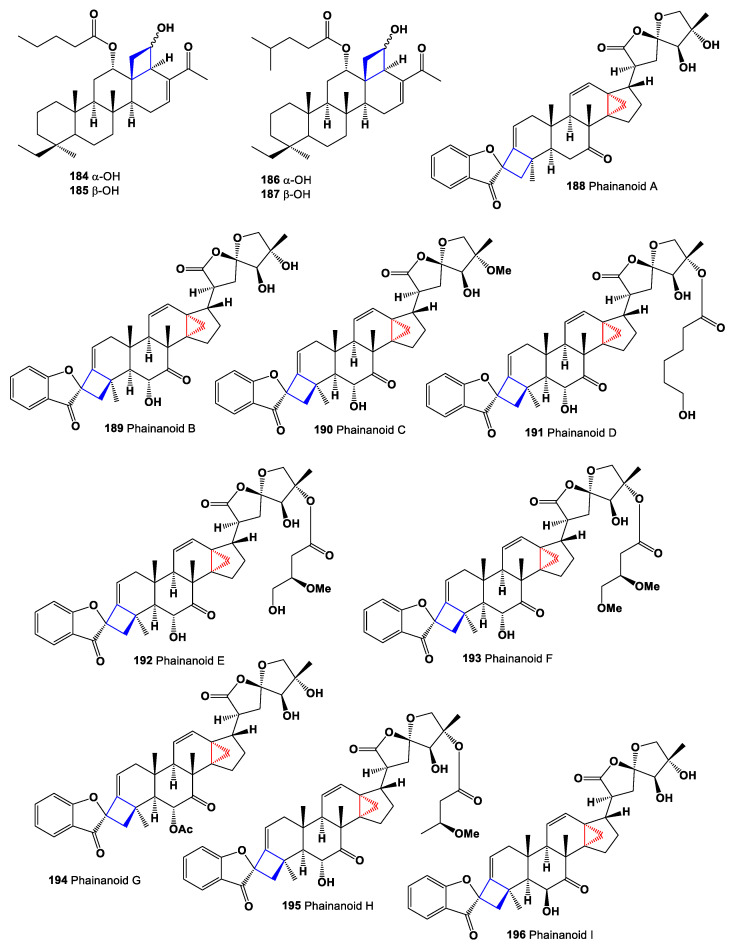
Bioactive cyclobutane-containing steroids and triterpenoids.

Scalarane sesterterpenoids 20,24-bishomoscalaranes, carteriofenones Е (**184**), F (**185**), G (**186**), and H (**187**) were obtained from the marine sponge *Carteriospongia foliascens,* collected from the South China Sea. These compounds represented rare, naturally occurring scalaranes with a cyclobutane ring [193]. Chemical structures **184**–**196** are shown in Figure 14, and their biological activity is shown in Table 13.

The shrub *Phyllanthus hainanensis*, which is endemic to the island of Hainan province of China, has been used in traditional Chinese medicine for over 1000 years, has great pharmaceutical potential to treat diseases such as cancer and diabetes, and is also used to prevent, and treat, chronic hepatitis B virus infection [194,195]. Several highly modified triterpenoids, with a new carbon skeleton by incorporating two unique motifs of a 4,5- and a 5,5-spirocyclic systems and containing cyclopropane and cyclobutene fragments, named phainanoids A (**188**), B (**189**), C (**190**), D (**191**), E (**192**), F (**193**), G (**194**), H (**195**), and I (**196**), have been determined in the extracts of the *Phyllanthus hainanensis* [196,197]. All compounds exhibited exceptionally potent immunosuppressive activities in vitro against the proliferation of T and B lymphocytes. The most potent one, phainanoid F, showed activities against the proliferation of T cells with IC_50_ value of 2 nM (positive control CsA = 14 nM) and B cells with IC_50_ value of <1.6 nM (CsA = 352.8 nM), which is about 7 and 221 times as active as CsA, respectively.

**Figure 15:**
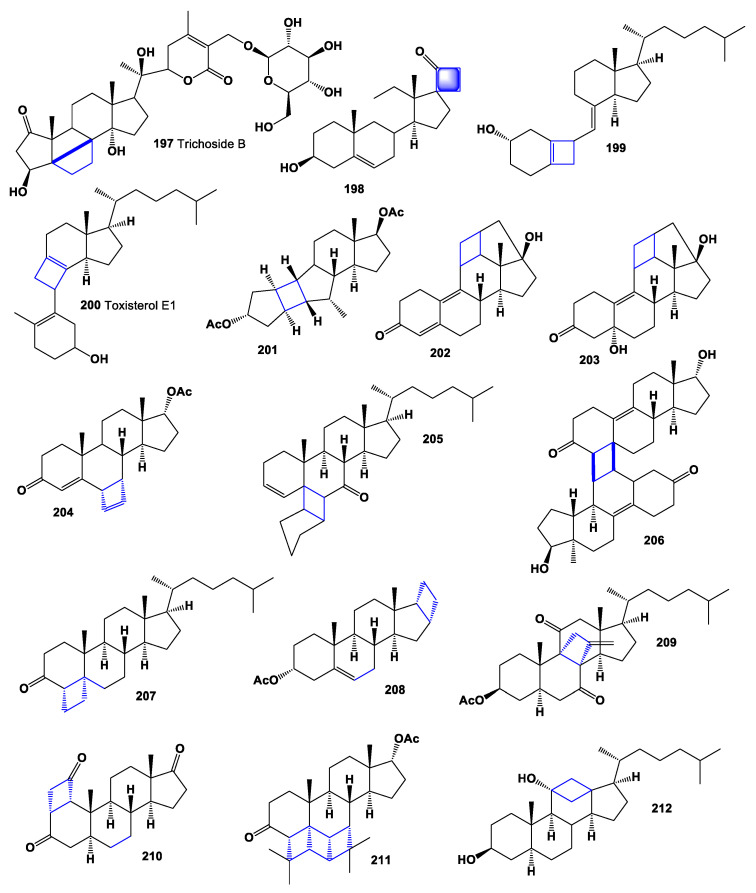
Bioactive natural and synthetic cyclobutane-containing steroids and triterpenoids.

Trichoside B (**197**, chemical structures **197**–**212** are shown in Figure 15, and their biological activity is shown in Table 14), withanolide glucoside, has been isolated from the n-butanolic fraction of the 75% methanolic extract of aerial parts of *Tricholepis eburnea* [198], and other unusual cyclobutene, containing secosteroid (**198**), was detected in oil from a pineal tropical plant *Sida cordata* (family Malvaceae), which is used to treat various diseases and ailments in many complementary and alternative medicine systems [199]. Studying the photoproducts obtained by photochemical processes of vitamin D, cyclobutane, containing vitamin D (**199**), was identified [200]. Toxisterol (**200**), as a minor transformation product of vitamin D2, has been found in various mushrooms [152].

A unique non-olefinic product containing a cyclobutane fragment (**201**) was obtained from 5,10-seco steroid containing Δ^1(10)^—and Δ^5(6)^ -double bonds in the AB ring during photochemical transformation [201]. The steroid altrenogest, a progestin of the 19-nortestosterone group, which is widely used in veterinary medicine to suppress or synchronize estrus in horses and pigs, using photolysis experiments gives two photoproducts: (**202**) and (**203**) [202].

In the chemistry of steroid hormones, the modification of the skeleton of natural steroids is used to obtain compounds with a narrower and more targeted spectrum of biological action, which makes it possible for their practical application. Among the many types of such transformed steroids, compounds containing an additional carbocycle are of great interest [203,204,205].

Photochemical [2 + 2]-cycloaddition is a common method for the construction of pentacyclic steroids and, in contrast to dark reactions, allows the introduction of a cyclobutane moiety anywhere in the steroid molecule. Several pentacyclic steroids, with an additional four-membered cycle (**204**–**212**), have been synthesized using various photochemical methods, while the biological activity of synthetic steroids has not been studied [18,204,205].

**Figure 16:**
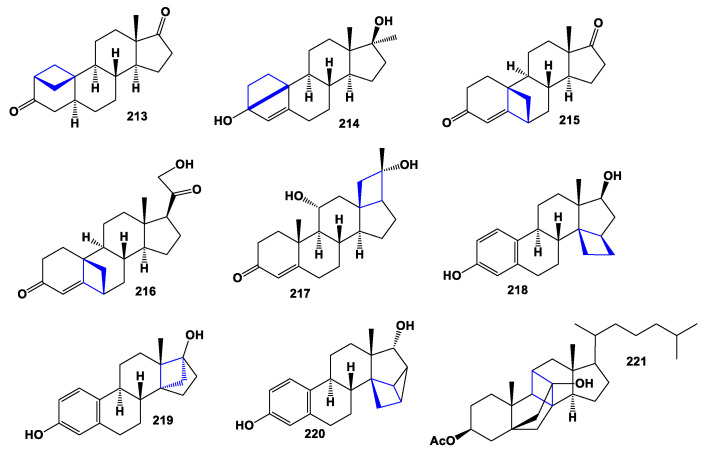
Bioactive synthetic steroids containing an additional 4-membered ring in the steroid molecule.

As a potent inhibitor of aromatase [206,207], 2,19-Methano-androstenedione (**213**) was synthesized, and the steroid (**214**) has a 3,9-carbon bridge like that of the steroid, trichoside B [208]. Two 6,19-cycloprogesterones (**215** and **216**) were synthesized from 11,19-epithiopregnane, and the end products showed increased affinity for glucocorticoid receptors [209]. Steroids (**218**–**221**), with a cyclobutane moiety anywhere in the steroid molecule, have been synthesized with the aim of finding bioactive anticancer agents [160,167,168,210]. Chemical structures **213**–**221** are shown in Figure 16, and their biological activity is shown in Table 15.

## 5. Miscellaneous Cyclosteroids and Triterpenoids Derived from Marine and Terrestrial Sources

**Figure 17:**
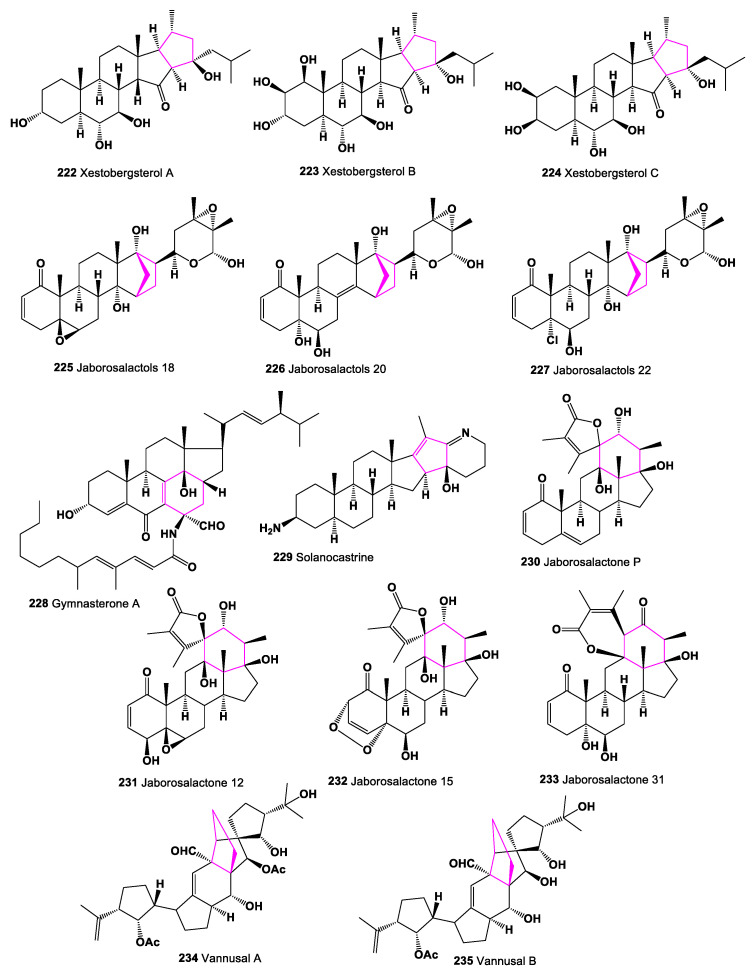
Bioactive steroids containing an additional 5- or 6-membered ring in molecule.

Two unique pentacyclic polyhydroxylated sterols (23S-16/S,23-cyclo-3α,6α,7φ8,23-tetrahydroxy-5α,14|9-cholestan-15-one, named xestobergsterol A (**222**), and 23S-16/3,23-cyclo-l/8,2/3,3α,6α,7|8,23-hexahydroxy-5α,14/3-cholestan-15-one, named xestobergsterol B (**223**)) have been found and identified from marine sponge *Xestospongia bergquistia* [211], and xestobergsterol C (**224**) was detected in the Okinawan marine sponge *Ircinia* sp. [212]. Chemical structures **222**–**235** are shown in Figure 17, and their biological activity is shown in Table 16.

Carbon-bridged steroids which were isolated from *Jaborosa bergii* presented a norbornane-type structure in ring D of the steroid nucleus (**225**–**227**), resulting from a carbon−carbon bond between C-15 and C-21. Jaborosalactols 18 (**225**) and 22 (**227**) have a 14α-hydroxy group while jaborosalactol 20 (**226**) contains 8,14-double bond [213].

The unusual cytotoxic steroid named gymnasterones A (**228**) was isolated from the microscopic fungus *Gymnascella dankaliensis,* associated with the sponge *Halichondria japonica* [214].

A steroidal alkaloid with a C-C linkage between C-16 and C-23, 3β-amino-16,23-cyclo-23β-hydroxy-5∝,16ξ,25β-22,26-epiminocholestan-17(20),22(N)-diene named solanocastrine (**229**) has been identified from extracts of the leaves of *Solanum capsicastrum* [215].

The spiranoid-γ-lactone steroid series have been found in lipid extracts in the genus Jaborosa. Interestingly, the first triterpenoid with a spiranoid-γ-lactone side chain was jaborosalactone P (**230**), which was collected over 30 years ago in extracts of *Jaborosa odonelliana* collected in Argentina [216]. Other related metabolites, such as jaborosalactone 12 (**231**), jaborosalactone 15 (**232**), and jaborosalactone 31 (**233**), were isolated from *Jaborosa odonelliana*, and jaborosalactone P was the major component in all samples collected in both spring and summer. In addition, jaborosalactone 31 (**230**) was found in extracts of all species studied, *J. rotacea*, *J. odonelliana*, *J. runcinata*, and *J. araucana* [217,218,219]. The triterpenes, named vannusals A (**234**) and B (**235**), with unusual skeletons, were obtained from the marine ciliate *Euplotes vannus* [220,221,222,223,224,225], and both compounds showed strong cytotoxic activity. Unusual 2,3-secofernane triterpenoid, alstonic acid B (**236**) has been isolated from *Alsonia scholaris* [226].

**Figure 18:**
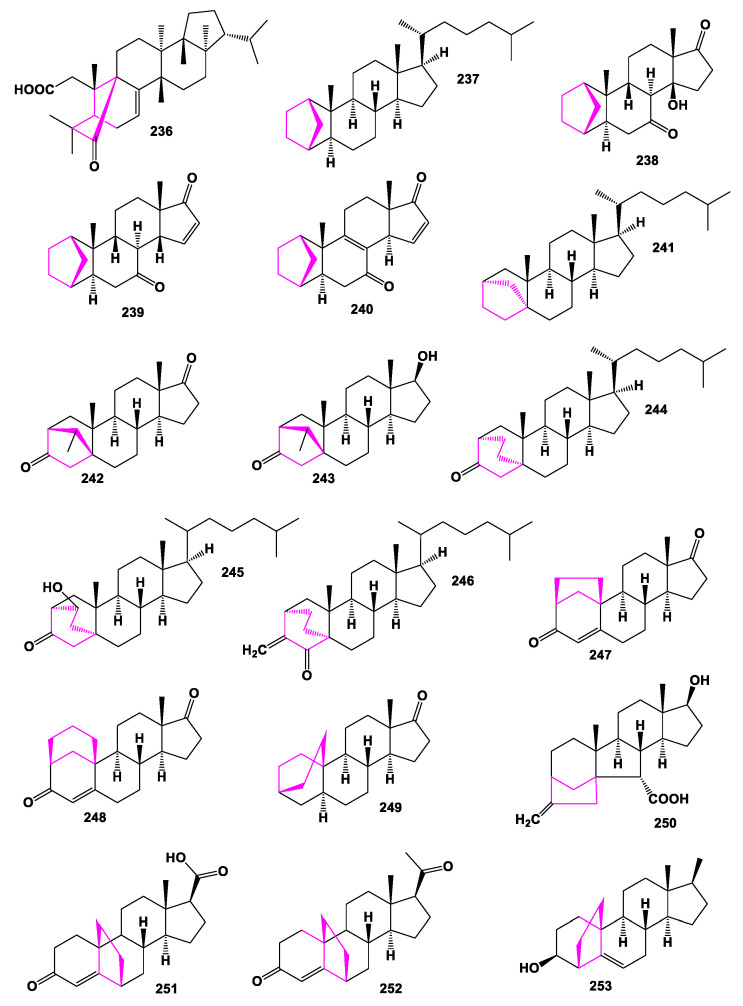
Bioactive cyclopentane- and cyclohexane-containing steroids.

**Figure 19:**
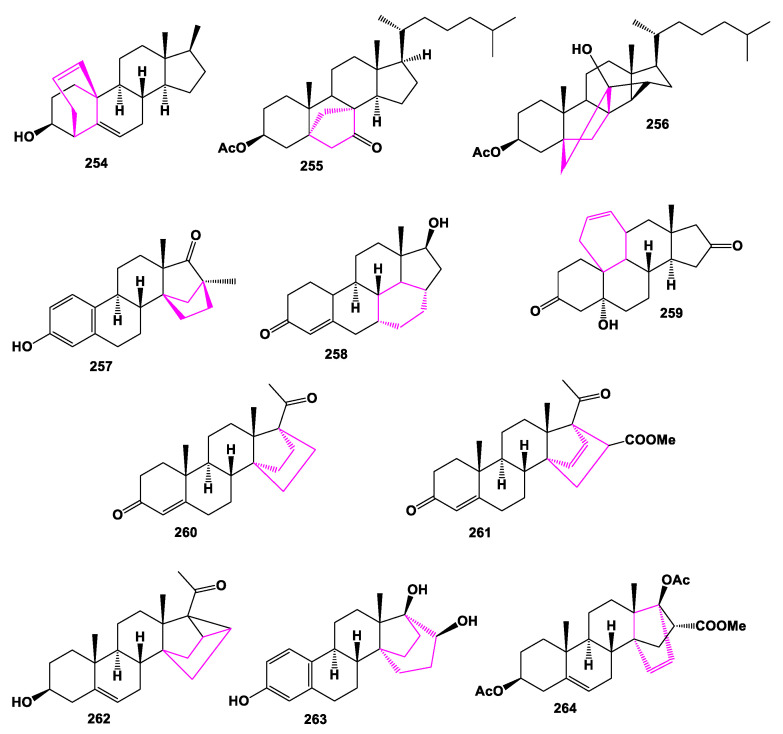
Bioactive synthethic steroids containing an additional 5- or 6-membered ring in molecule.

Several steroids (**237**–**264**), containing an additional 5- or 6-membered ring (s) in the steroid molecule, have been synthesized in various laboratories and demonstrate a wide range of biological activities [18,160,161,164,167,168,210,227,228,229,230,231,232], and their structures are shown in Figure 18 and Figure 19. Their pharmacological profile is presented in Table 16 and Table 17.

**Figure 20:**
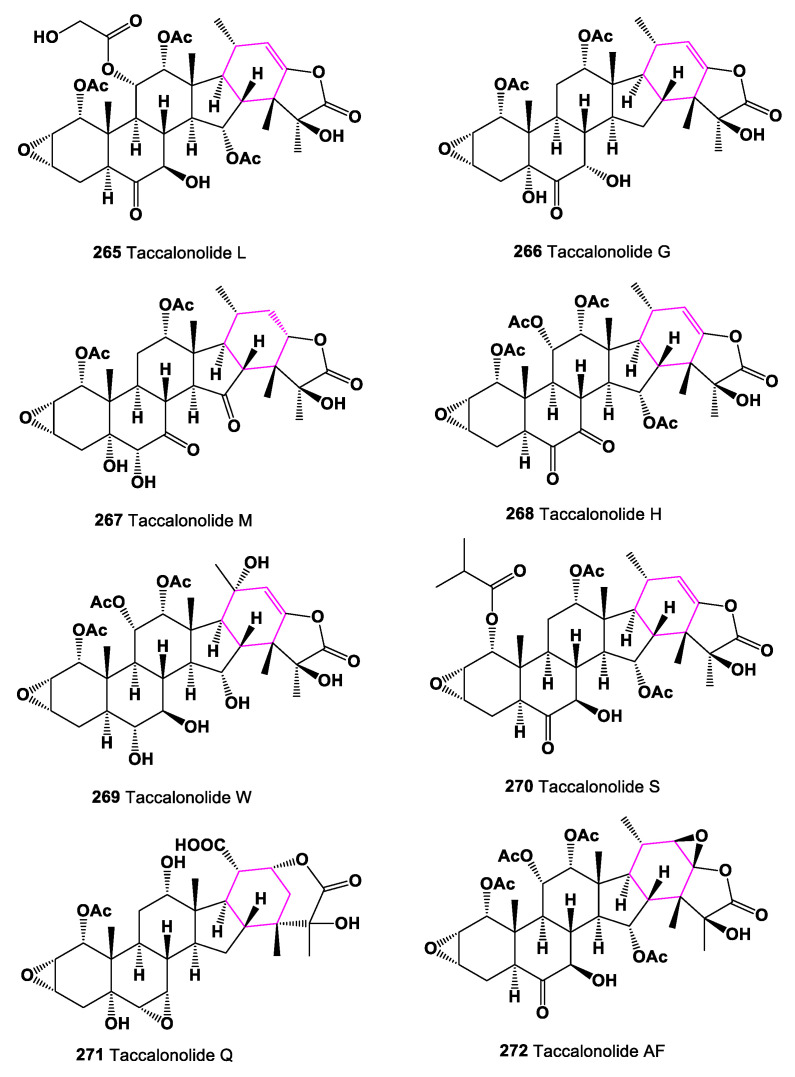
Bioactive steroids containing additional 6-membered ring in molecule.

Carbon-bridged steroids, called taccalonolides (**265**–**271**), are a class of microtubule-stabilizing agents that exhibit selective cancer-fighting properties [233]. Tacca species are known to contain highly oxygenated ixocarpalactone-type steroids, with an additional ring formed by a carbon–carbon bond between C-16 and C-24, taccalonolide A being the first example of these compounds [120]. Chemical structures **265**–**272** are shown in Figure 20, and their biological activity is shown in Table 18. Carbon-bridged steroids, related to taccalonolide A, were isolated from *Tacca plantaginea*, *Tacca subflaellata*, and the Vietnamese plant *Tacca paxiana* [234,235,236,237,238]. Taccalonolides AF (**272**) and AJ (**273**), showing antiproliferative properties, were isolated from a fraction of an ethanol extract of *T. plantaginea* [239], and a carbon-bridged steroid, named physanolide A (**274**), with an unprecedented skeleton containing a seven-membered ring was isolated from *Physalis angulate* [240].

**Figure 21:**
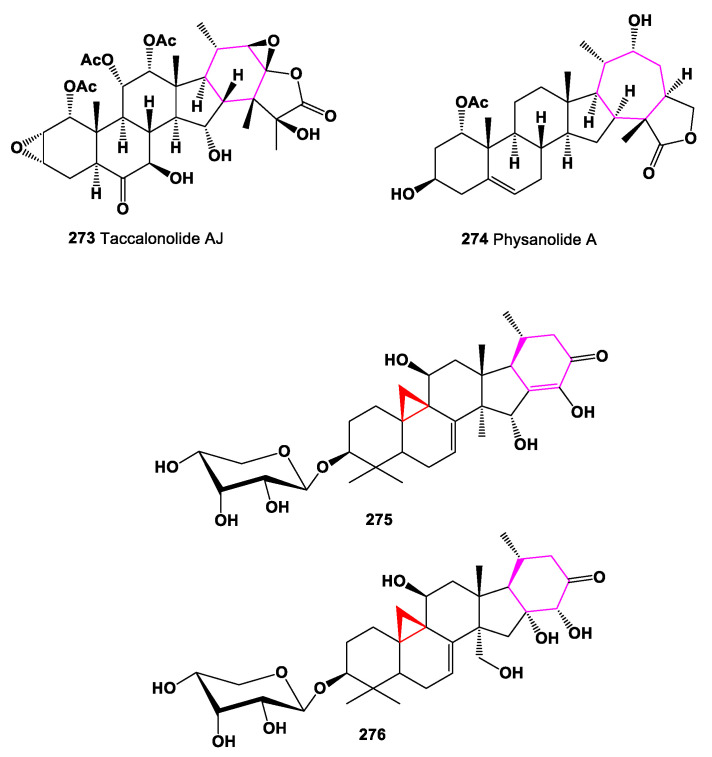
Bioactive steroids containing additional 6- or 7-membered ring in molecule.

Trinor-cycloartane glycosides, 15α-hydroxy-16-dehydroxy-16(24)-en-foetidinol-3-*O*-β-d-xylopyranoside (**275**) and 28-hydroxy-foetidinol-3-*O*-β-d-xylopyranoside (**276**) were isolated from the butanol fraction of the roots of *Cimicifuga foetida* [241]. Chemical structures **273**–**276** are shown in Figure 21, and their biological activity is shown in Table 18.

## 6. Comparison of Biological Activities of Natural and Synthetic CBS and Triterpenoids

It is known that the chemical structure of both natural and synthetic molecules predetermines biological activity, which makes it possible to analyze the structure-activity relationships (SAR). Such a wise idea was first proposed by Brown and Fraser more than 150 years ago, in 1868 [242]; although, according to other sources, SAR originates from the field of toxicology, according to which Cros, in 1863, determined the relationship between the toxicity of primary aliphatic alcohols and their solubility in water [243]. More than 30 years later, Richet in 1893 [244], Meyer in 1899 [245], and Overton in 1901 [246] separately found a linear correlation between lipophilicity and biological effects. By 1935, Hammett [247,248] presented a method of accounting for the effect of substituents on reaction mechanisms using an equation that considered two parameters, namely the substituent constant and the reaction constant. Complementing Hammett’s model, Taft proposed, in 1956, an approach for separating the polar, steric, and resonance effects of substituents in aliphatic compounds [249]. Combining all previous developments, Hansch and Fujita laid out the mechanistic basis for the development of the QSAR method [250], and the linear Hansch equation, and Hammett’s electronic constants, are detailed in the book by Hansch and Leo published in 1995 [251].

Some well-known computer programs can, with some degree of reliability, estimate the pharmacological activity of organic molecules isolated from natural sources or synthesized compounds [252,253,254]. It is known that classical SAR methods are based on the analysis of (quantitative) structure-activity relationships for one or more biological activities, using organic compounds belonging to the same chemical series as the training set [255].

Computer program PASS, which has been continuously updating and improving for the past thirty years [256], is based on the analysis of a heterogeneous training set included information about more than 1.3 million known biologically active compounds with data on ca. 10,000 biological activities [257,258]. Chemical descriptors implemented in PASS, which reflect the peculiarities of ligand-target interactions, and the original realization of the Bayesian approach for elucidation of structure-activity relationships provides the average accuracy, and predictivity, for several thousand biological activities equal to about 96% [259,260]. In several comparative studies, it was shown that PASS outperforms, in predictivity, some other recently developed methods for the estimation of biological activity profiles [261,262,263]. Freely available via the Internet, PASS Online web-service [264] is used by more than thirty thousand researchers from almost a hundred countries to determine the most promising biological activities for both natural and synthetic compounds [258,259,260,265]. To reveal the hidden pharmacological potential of the natural substances, we are successfully using PASS for the past fifteen years [266,267,268,269,270].

In the current study, we obtained PASS predictions for about three hundred steroids and triterpenoids produced by different living organisms. PASS estimates are presented as Pa values, which correspond to the probability of belonging to a class of “actives” for each predicted biological activity. The higher the Pa value is, the higher the confidence that the experiment will confirm the predicted biological activity [260].

### 6.1. Antitumor Activity of Cyclopropane-Containing CBS and Triterpenoids

Analyzing the data obtained using the PASS of natural cyclopropane containing steroids and triterpenoids, it can be stated that, out of 102 lipid molecules (**1**–**102**, see Figure 1, Figure 2, Figure 3, Figure 4, Figure 5, Figure 6 and Figure 7 and Table 1, Table 2, Table 3, Table 4, Table 5, Table 6 and Table 7), only 27 showed antitumor activity with a reliability of more than 90 percent, with two steroidal glycosides, (**25**) and (**41**), showed antitumor activity with more than 99% confidence. Thus, PASS has confirmed the cytotoxic properties of these steroids, which have been determined experimentally. Other sterols and triterpenoids, with a cyclopropane ring, demonstrated weak to moderate antitumor activity with 70 to 90 percent confidence.

**Figure 22:**
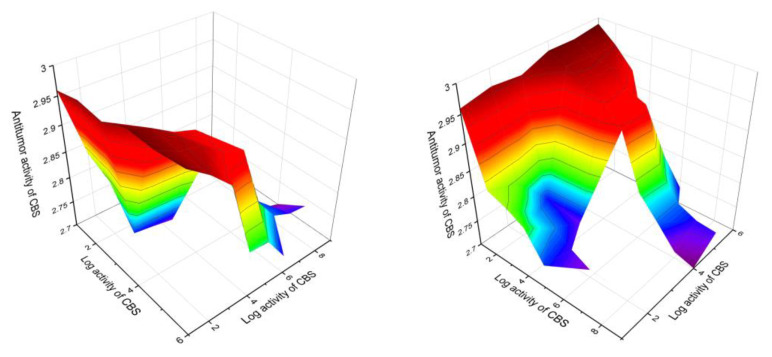
The 3D graph (X and Y views) shows the predicted and calculated antitumor activity of carbon- bridged steroids (CBS) with a cyclopropane ring in the side chain (compound numbers: **103**, **105**, **112**, **118**, **119** and **120**) showing the highest degree of confidence, more than 91%. These steroids derived from marine sponges *Petrosia weinbergi*, *Xestospongia* sp., *Poecillastra compressa*, and *Tethya* sp., and can be used in clinical medicine as potential agents with strong antitumor activity.

Among sterols and triterpenoids with a cyclopropane ring in the side chain, compounds were also found that demonstrate antitumor activity with a confidence level of more than 90 percent. These are steroids (**103**, 91.1%), (**105**, 93.4%), (**112**, 92.2%), (**118**, 96.3%), (**119**, 96.0%), and (**120**, 97.5%), which were isolated from the marine sponges *Petrosia weinbergi*, *Xestospongia* sp., *Poecillastra compressa*, and *Tethya* sp. A 3D graph of the predicted antitumor and related activities is shown in Figure 22.

**Figure 23:**
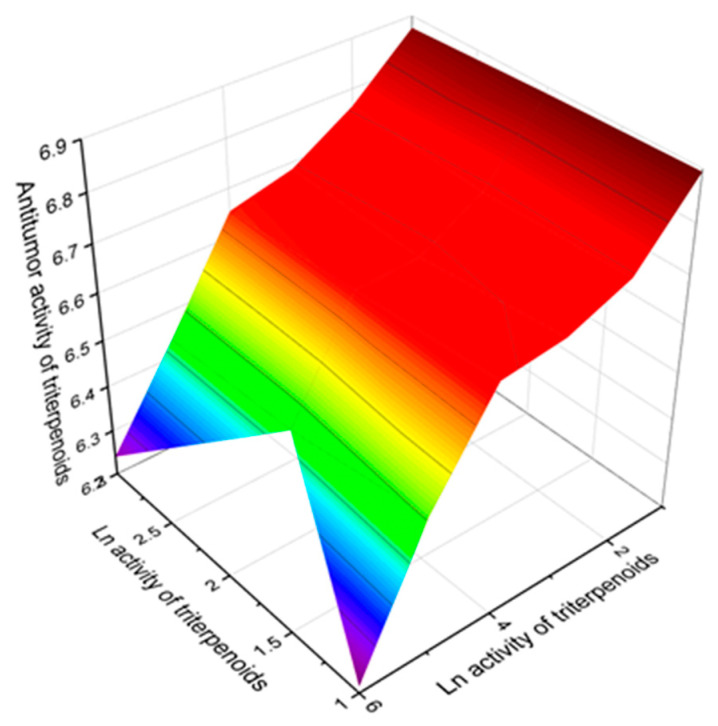
The 3D graph shows the predicted and calculated antitumor and related activities of cyclopropane-containing triterpenoid saponins (compound numbers: **146**, **147,** and **148**) showing the highest degree of confidence, more than 96%, which were isolated from the leaves and flowers extracts of *Verbesina virginica*, and can be used in clinical medicine as potential agents with strong antitumor activity.

Triterpenoid saponins, (**146**, 98.7%), (**147**, 98.0%), and (**148**, 96.9%), containing the cyclopropane ring at position 15:27, were isolated from the leaves and flowers extracts of *Verbesina virginica*, demonstrating the highest degree of confidence—more than 96%. A 3D graph of the predicted antitumor and related activities is shown in Figure 23.

### 6.2. Antitumor Activity of Cyclobutane-Containing CBS and Triterpenoids

**Figure 24:**
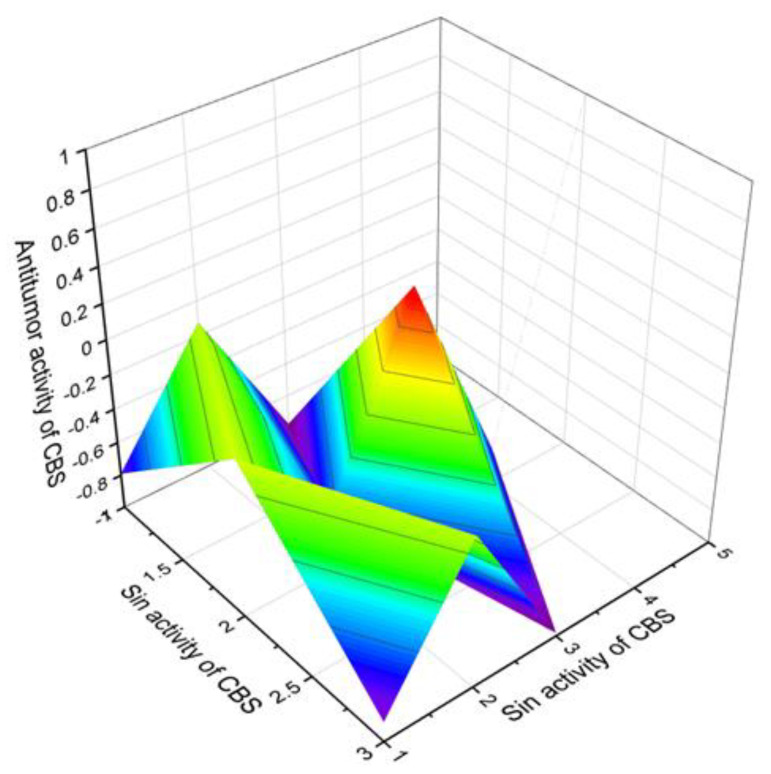
The 3D graph shows the predicted and calculated antitumor and related activities of cyclobutane-containing steroids (compound numbers: **197**, **206,** and **214**) showing the highest degree of confidence, more than 90%.

Cyclobutane containing steroids and triterpenoids (**165**–**221**), isolated from natural sources as well as semi- and synthetic compounds, were also analyzed using PASS. Most of these lipid molecules showed moderate antitumor activity with 70 to 90 percent confidence, and only three, (**197**, 92.9%), (**206**, 90.8%), and (**214**, 90.9%), steroids showed antitumor activity with more than 90% confidence. A 3D graph of the predicted antitumor and related activities is shown in Figure 24.

The withanolide glucoside named trichoside B (**197**) is of type A-nor-sterols, and was isolated from the methanolic extract of aerial parts of *Tricholepis eburnea,* which is native to Afghanistan, compound (**206**) is a testosterone derivative dimer, and the steroid (**214**) contains a cyclobutane ring in ring A of the steroid.

### 6.3. Miscellaneous Cyclosteroids and Triterpenoids

**Figure 25:**
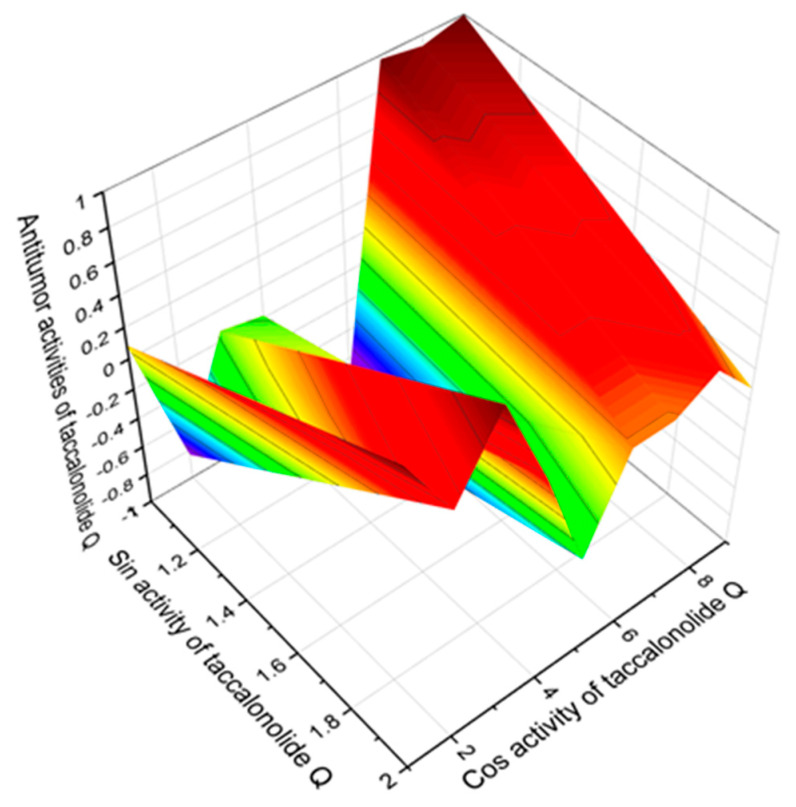
The 3D graph shows the predicted and calculated pharmacological activities of taccalonolide Q (**271**). Taccalonolide Q, similar to other taccalonolides, is a class of highly acetoxylated pentacyclic steroids containing 28 carbons, known microtubule stabilizing cytotoxic agents isolated from the genus Tacca that have selective anti- cancer properties. Taccalonolide Q has a C2–C3 epoxide group and an enol-γ-lactone fused with the unique E ring. In addition to total antineoplastic activity with a high confidence level of 93%, taccalonolide Q demonstrates selective activity against renal cancer, sarcoma, pancreatic cancer, lymphocytic leukemia, myeloid leukemia, and genitourinary cancer.

Miscellaneous cyclosteroids and triterpenoids (**222**–**276**, see Figure 17, Figure 18, Figure 19, Figure 20 and Figure 21, and Table 16, Table 17 and Table 18) make up one-fifth of all compounds presented in this work. Two-thirds of lipid molecules demonstrate moderate activity, and seventeen compounds show strong antitumor activity with a confidence level of more than 90%, and the triterpenoid called taccalonolide Q (**271**) has the widest spectrum of biological activities among antitumor agents. A 3D graph of the predicted antitumor activities is shown in Figure 25. The data we obtained using PASS are supported by the data just published by Peng and colleagues, which shows a wide range of biological activities of taccalonolides [271].

## 7. Conclusions

This review focuses on a rare group of carbon-bridged steroids (CBS) and triterpenoids found in lipid extracts from various natural sources such as green, yellow-green, and red algae, sea sponges, soft corals, ascidians, starfish, and other marine invertebrates. These compounds are also found in amoebas, fungi, fungal endophytes, and plants. There are 276 steroids and triterpenoids presented in this review, which demonstrate a wide range of biological activities, but the most pronounced antitumor profile. This review summarizes biological activities as experimentally obtained and published in the open press, as well as by using the extensive PASS program. We must state that two-thirds of carbon-bridged steroids and triterpenoids show moderate activity levels with 70 to 90% confidence, and only one-third of these lipids show strong antitumor activity with more than 90% confidence. All lipid material presented is divided into four groups, which include: (a) CBS and triterpenoids containing a cyclopropane moiety; (b) CBS and triterpenoids with cyclopropane ring in the side chain; (c) CBS and triterpenoids containing a cyclobutane moiety; (d) CBS and triterpenoids containing cyclopentane, cyclohexane, or cycloheptane moieties. The most important conclusion shows that some CBS and triterpenoids from different lipid groups demonstrate selective action on different types of tumor cells, such as renal cancer, sarcoma, pancreatic cancer, prostate cancer, lymphocytic leukemia, myeloid leukemia, liver cancer, and genitourinary cancer with different degree of reliability.

## Figures and Tables

**Table 1:** Biological activities of cyclopropane-containing carbon-bridged steroids.

No.	Antitumor & Related Activity, (Pa) *	Lipid Metabolism Regulators, (Pa) *	Additional Predicted Activity, (Pa) *
**1**	Antineoplastic (0.915) Apoptosis agonist (0.892) Antineoplastic (liver cancer) (0.822) Chemopreventive (0.776) Cytoprotectant (0.611) Prostate cancer treatment (0.557) Antimetastatic (0.528)	Anti-hypercholesterolemic (0.900) Hypolipemic (0.897) Atherosclerosis treatment (0.690)	Anti-osteoporotic (0.861) Anti-eczematic (0.850) Immunosuppressant (0.744) Antiparkinsonian, rigidity relieving (0.720) Anti-inflammatory (0.706)
**2**	Chemopreventive (0.968) Apoptosis agonist (0.879) Antineoplastic (0.867) Cytoprotectant (0.645) Antimetastatic (0.578)	Hypolipemic (0.874) Anti-hypercholesterolemic (0.649) Cholesterol synthesis inhibitor (0.614) Lipid metabolism regulator (0.598) Atherosclerosis treatment (0.594)	Anti-eczematic (0.889) Anti-inflammatory (0.860) Antifungal (0.821) Immunosuppressant (0.742) Anti-psoriatic (0.720)
**3**	Chemopreventive (0.923) Antineoplastic (0.863) Cytoprotectant (0.704) Antimetastatic (0.655) Antineoplastic (liver cancer) (0.608) Anticarcinogenic (0.553) Proliferative diseases treatment (0.551) Antineoplastic (pancreatic cancer) (0.544)	Hypolipemic (0.879) Anti-hypercholesterolemic (0.847) Cholesterol synthesis inhibitor (0.705) Atherosclerosis treatment (0.674)	Anti-eczematic (0.900) Anti-inflammatory (0.843) Antifungal (0.806) Antipruritic (0.776) Immunosuppressant (0.750) Anti-psoriatic (0.744) Anti-osteoporotic (0.716)
**4**	Chemopreventive (0.857) Antineoplastic (0.839) Apoptosis agonist (0.799) Cytoprotectant (0.646) Antimetastatic (0.623) Antineoplastic (pancreatic cancer) (0.514)	Hypolipemic (0.883) Anti-hypercholesterolemic (0.739) Cholesterol synthesis inhibitor (0.731) Atherosclerosis treatment (0.665)	Anti-eczematic (0.871) Anti-fungal (0.823) Anti-inflammatory (0.805) Anti-osteoporotic (0.707) Anti-psoriatic (0.683)
**5**	Chemopreventive (0.842) Antineoplastic (0.840) Cytoprotectant (0.680) Antimetastatic (0.647) Proliferative diseases treatment (0.555) Prostatic (benign) hyperplasia treatment (0.540) Antineoplastic (pancreatic cancer) (0.528)	Hypolipemic (0.857) Anti-hypercholesterolemic (0.788) Cholesterol synthesis inhibitor (0.697) Atherosclerosis treatment (0.663)	Anti-eczematic (0.880) Anti-inflammatory (0.808) Anti-fungal (0.781) Anti-psoriatic (0.719)
**6**	Chemopreventive (0.866) Antineoplastic (0.715)	Hypolipemic (0.703) Cholesterol synthesis inhibitor (0.521)	Antifungal (0.878) Anti-inflammatory (0.771)
**7**	Chemopreventive (0.849) Antineoplastic (0.766)	Hypolipemic (0.676) Cholesterol synthesis inhibitor (0.554)	Antifungal (0.836) Anti-inflammatory (0.737)
**8**	Chemopreventive (0.713) Antineoplastic (0.690) Apoptosis agonist (0.584)	Hypolipemic (0.742) Atherosclerosis treatment (0.644) Cholesterol synthesis inhibitor (0.593)	Antifungal (0.850) Anti-inflammatory (0.759)
**9**	Chemopreventive (0.949) Apoptosis agonist (0.822) Antineoplastic (0.801) Antimetastatic (0.558)	Hypolipemic (0.788) Cholesterol synthesis inhibitor (0.572) Atherosclerosis treatment (0.508)	Antifungal (0.884) Anti-inflammatory (0.814)
**10**	Chemopreventive (0.765) Antineoplastic (0.701)	Hypolipemic (0.711) Cholesterol synthesis inhibitor (0.571)	
**11**	Chemopreventive (0.836) Apoptosis agonist (0.763) Antineoplastic (0.755)	Hypolipemic (0.744) Cholesterol synthesis inhibitor (0.546) Atherosclerosis treatment (0.511)	Anti-eczematic (0.701)
**12**	Chemopreventive (0.938) Antineoplastic (0.804) Apoptosis agonist (0.623)	Hypolipemic (0.736) Atherosclerosis treatment (0.641) Cholesterol synthesis inhibitor (0.575)	Hepatoprotectant (0.900)
**13**	Chemopreventive (0.928) Antineoplastic (0.812) Apoptosis agonist (0.763)	Hypolipemic (0.800) Atherosclerosis treatment (0.609) Cholesterol synthesis inhibitor (0.532)	Hepatoprotectant (0.861)
**14**	Chemopreventive (0.956) Apoptosis agonist (0.832) Antineoplastic (0.825)	Hypolipemic (0.847) Atherosclerosis treatment (0.657) Cholesterol synthesis inhibitor (0.568)	Hepatic disorders treatment (0.898)
**15**	Chemopreventive (0.935) Apoptosis agonist (0.821) Antineoplastic (0.789)	Hypolipemic (0.796) Atherosclerosis treatment (0.623) Cholesterol synthesis inhibitor (0.618)	Hepatoprotectant (0.823)
**16**	Chemopreventive (0.944) Apoptosis agonist (0.808) Antineoplastic (0.795) Anticarcinogenic (0.628)	Hypolipemic (0.842) Cholesterol synthesis inhibitor (0.714) Atherosclerosis treatment (0.708)	Hepatoprotectant (0.872) Antifungal (0.831) Anti-inflammatory (0.823)
**17**	Apoptosis agonist (0.864) Antineoplastic (0.841) Chemopreventive (0.824) Antimetastatic (0.610) Antineoplastic (melanoma) (0.570) Proliferative diseases treatment (0.537) Bone diseases treatment (0.529) Antineoplastic (pancreatic cancer) (0.516)	Hypolipemic (0.816) Atherosclerosis treatment (0.665) Cholesterol synthesis inhibitor (0.579)	Anti-eczematic (0.865) Antifungal (0.819)
**18**	Chemopreventive (0.909) Apoptosis agonist (0.873) Antineoplastic (0.847) Antimetastatic (0.629)	Hypolipemic (0.894) Atherosclerosis treatment (0.670) Cholesterol synthesis inhibitor (0.625) Anti-hypercholesterolemic (0.622)	Hepatic disorders treatment (0.842) Antiinflammatory (0.839) Antieczematic (0.831) Antifungal (0.809)

* Only activities with Pa > 0.5 are shown.

**Table 2:** Biological activities of carbon-bridged steroids.

No.	Antitumor & Related Activity, (Pa) *	Lipid Metabolism Regulators, (Pa) *	Additional Predicted Activity, (Pa) *
**19**	Chemopreventive (0.858) Antineoplastic (0.815) Apoptosis agonist (0.811) Antimetastatic (0.620)	Hypolipemic (0.863) Cholesterol synthesis inhibitor (0.536)	Anti-eczematic (0.809) Anti-ulcerative (0.765)
**20**	Chemopreventive (0.923) Apoptosis agonist (0.847) Antineoplastic (0.837) Cytoprotectant (0.652) Antimetastatic (0.634)	Hypolipemic (0.861) Atherosclerosis treatment (0.624) Cholesterol synthesis inhibitor (0.613)	Antieczematic (0.837) Antiinflammatory (0.833) Antifungal (0.829)
**21**	Antineoplastic (0.894) Chemopreventive (0.851) Apoptosis agonist (0.810) Antimetastatic (0.589) Cytoprotectant (0.576)	Hypolipemic (0.867) Atherosclerosis treatment (0.512)	Anti-eczematic (0.850) Anti-inflammatory (0.755)
**22**	Chemopreventive (0.959) Antineoplastic (0.886) Apoptosis agonist (0.858) Cytoprotectant (0.701) Antineoplastic (liver cancer) (0.641) Antimetastatic (0.607) Proliferative diseases treatment (0.554) Prostate cancer treatment (0.510)	Hypolipemic (0.877) Atherosclerosis treatment (0.676) Anti-hypercholesterolemic (0.609) Cholesterol synthesis inhibitor (0.568) Lipid metabolism regulator (0.553)	Hepatic disorders treatment (0.921) Anti-eczematic (0.877) Anti-inflammatory (0.872) Anti-psoriatic (0.808)
**23**	Chemopreventive (0.967) Antineoplastic (0.884) Apoptosis agonist (0.881) Cytoprotectant (0.638) Antimetastatic (0.615)	Hypolipemic (0.881) Atherosclerosis treatment (0.654) Cholesterol synthesis inhibitor (0.568) Lipid metabolism regulator (0.544)	Anti-eczematic (0.888) Anti-inflammatory (0.827) Antifungal (0.800) Anti-psoriatic (0.739)
**24**	Chemopreventive (0.952) Apoptosis agonist (0.897) Antineoplastic (0.857) Cytoprotectant (0.677) Antimetastatic (0.657) Anticarcinogenic (0.561) Antineoplastic (liver cancer) (0.552) Proliferative diseases treatment (0.538) Antineoplastic (pancreatic cancer) (0.537)	Hypolipemic (0.900) Atherosclerosis treatment (0.689) Cholesterol synthesis inhibitor (0.671) Anti-hypercholesterolemic (0.662) Lipid metabolism regulator (0.529)	Anti-eczematic (0.879) Anti-psoriatic (0.709)
**25**	Chemopreventive (0.991) Antineoplastic (0.915) Apoptosis agonist (0.879) Anticarcinogenic (0.787) Proliferative diseases treatment (0.735) Antimetastatic (0.579) Antineoplastic (sarcoma) (0.533)	Hypolipemic (0.825) Anti-hypercholesterolemic (0.816) Atherosclerosis treatment (0.669)	Hepatoprotectant (0.987) Antifungal (0.893) Anti-inflammatory (0.882)
**26**	Chemopreventive (0.881) Antineoplastic (0.854) Apoptosis agonist (0.825) Antimetastatic (0.544)	Hypolipemic (0.833) Cholesterol synthesis inhibitor (0.821) Anti-hypercholesterolemic (0.791) Lipoprotein disorders treatment (0.717)	Antifungal (0.867) Anti-eczematic (0.830) Anti-inflammatory (0.804)
**27**	Antineoplastic (0.867) Apoptosis agonist (0.742) Chemopreventive (0.707) Cytoprotectant (0.656) Proliferative diseases treatment (0.606) Antimetastatic (0.565) Chemoprotective (0.558) Antineoplastic (pancreatic cancer) (0.544) Anticarcinogenic (0.541)	Hypolipemic (0.698) Atherosclerosis treatment (0.594) Anti-hypercholesterolemic (0.550) Cholesterol synthesis inhibitor (0.521)	Antieczematic (0.886) Hepatoprotectant (0.861) Antipsoriatic (0.714)
**28**	Antineoplastic (0.875) Chemopreventive (0.780) Apoptosis agonist (0.768) Proliferative diseases treatment (0.687) Cytoprotectant (0.685) Anticarcinogenic (0.639) Antimetastatic (0.590) Antineoplastic (pancreatic cancer) (0.549)	Anti-hypercholesterolemic (0.714) Hypolipemic (0.698) Antipruritic (0.639) Atherosclerosis treatment (0.582) Cholesterol synthesis inhibitor (0.576)	Hepatoprotectant (0.858) Immunosuppressant (0.751) Hepatic disorders treatment (0.686)
**29**	Antineoplastic (0.881) Chemopreventive (0.791) Apoptosis agonist (0.669) Proliferative diseases treatment (0.666) Anticarcinogenic (0.657) Cytoprotectant (0.627) Chemoprotective (0.565) Antimetastatic (0.559) Antineoplastic (pancreatic cancer) (0.547)	Anti-hypercholesterolemic (0.738) Hypolipemic (0.707) Cholesterol synthesis inhibitor (0.559) Atherosclerosis treatment (0.539)	Anti-eczematic (0.898) Hepatoprotectant (0.866)
**30**	Antineoplastic (0.814) Apoptosis agonist (0.801) Chemopreventive (0.782) Cytoprotectant (0.604) Antineoplastic (pancreatic cancer) (0.565) Antimetastatic (0.526)	Hypolipemic (0.830) Cholesterol synthesis inhibitor (0.679) Anti-hypercholesterolemic (0.618) Atherosclerosis treatment (0.546)	Anti-eczematic (0.847) Antiinflammatory (0.794) Antifungal (0.789) Immunosuppressant (0.733) Antiosteoporotic (0.727)
**31**	Antineoplastic (0.797) Apoptosis agonist (0.766) Chemopreventive (0.762) Cytoprotectant (0.585) Antineoplastic (pancreatic cancer) (0.559) Prostatic (benign) hyperplasia treatment (0.519) Antimetastatic (0.516)	Hypolipemic (0.742) Cholesterol synthesis inhibitor (0.583)	Anti-eczematic (0.831) Antiinflammatory (0.771) Antifungal (0.751)
**32**	Antineoplastic (0.803) Apoptosis agonist (0.719) Chemopreventive (0.696) Prostatic (benign) hyperplasia treatment (0.599) Antineoplastic (pancreatic cancer) (0.538)		Erythropoiesis stimulant (0.743) Diuretic (0.629) Anesthetic general (0.611)
**33**	Chemopreventive (0.889) Antineoplastic (0.837) Apoptosis agonist (0.751) Cytoprotectant (0.720) Antineoplastic (pancreatic cancer) (0.563) Antineoplastic enhancer (0.558) Antimetastatic (0.543)	Hypolipemic (0.752) Anti-hypercholesterolemic (0.669) Cholesterol synthesis inhibitor (0.607) Atherosclerosis treatment (0.527)	
**34**	Apoptosis agonist (0.854) Antineoplastic (0.846) Chemopreventive (0.831) Cytoprotectant (0.687) Antimetastatic (0.635) Proliferative diseases treatment (0.577) Antineoplastic (pancreatic cancer) (0.559)	Hypolipemic (0.875) Anti-hypercholesterolemic (0.681) Atherosclerosis treatment (0.639) Cholesterol synthesis inhibitor (0.599)	Anti-eczematic (0.900)
**35**	Antineoplastic (0.816) Apoptosis agonist (0.799) Chemopreventive (0.738) Cytoprotectant (0.661) Antimetastatic (0.624) Proliferative diseases treatment (0.580) Antineoplastic (pancreatic cancer) (0.547)	Hypolipemic (0.852) Atherosclerosis treatment (0.623) Anti-hypercholesterolemic (0.594) Cholesterol synthesis inhibitor (0.592)	Anti-eczematic (0.880)
**36**	Antineoplastic (0.886) Chemopreventive (0.819) Apoptosis agonist (0.769) Antimetastatic (0.630) Antineoplastic (renal cancer) (0.593) Antineoplastic (lymphocytic leukemia) (0.525) Prostate cancer treatment (0.511)	Hypolipemic (0.795)	Diabetic neuropathy treatment (0.884) Antidiabetic symptomatic (0.778)

* Only activities with Pa > 0.5 are shown.

**Table 3:** Biological activities of carbon-bridged steroids.

No.	Antitumor & Related Activity, (Pa) *	Lipid Metabolism Regulators, (Pa) *	Additional Predicted Activity, (Pa) *
**37**	Antineoplastic (0.877) Apoptosis agonist (0.771) Antiparasitic (0.631) Chemopreventive (0.629) Antimetastatic (0.577)		Spasmolytic, urinary (0.696)
**38**	Antineoplastic (0.852) Apoptosis agonist (0.785) Chemopreventive (0.665) Antimetastatic (0.578)		Spasmolytic, urinary (0.671)
**39**	Antineoplastic (0.898) Chemopreventive (0.849) Apoptosis agonist (0.823) Antimetastatic (0.554)	Hypolipemic (0.581)	
**40**	Antineoplastic (0.785) Chemopreventive (0.715) Apoptosis agonist (0.588)	Hypolipemic (0.556)	
**41**	Chemopreventive (0.994) Antineoplastic (0.910) Apoptosis agonist (0.826)	Hypolipemic (0.651)	
**42**	Antineoplastic (0.775) Apoptosis agonist (0.716) Chemopreventive (0.626) Antimetastatic (0.583)		Alzheimer’s disease treatment (0.831) Neurodegenerative diseases treatment (0.818) Antiparkinsonian (0.556)
**43**	Antineoplastic (0.842) Apoptosis agonist (0.575) Antimetastatic (0.505)		
**44**	Antineoplastic (0.860) Apoptosis agonist (0.851) Chemopreventive (0.797) Antimetastatic (0.585) Antineoplastic enhancer (0.571) Antineoplastic (sarcoma) (0.548)	Hypolipemic (0.809)	
**45**	Antineoplastic (0.857) Chemopreventive (0.731) Apoptosis agonist (0.702) Antimetastatic (0.589)	Hypolipemic (0.787)	
**46**	Antineoplastic (0.921) Apoptosis agonist (0.822) Chemopreventive (0.748) Antimetastatic (0.607) Antineoplastic (renal cancer) (0.538)	Hypolipemic (0.590) Cholesterol synthesis inhibitor (0.525)	
**47**	Chemopreventive (0.910) Antineoplastic (0.892) Apoptosis agonist (0.887) Anticarcinogenic (0.554) Antineoplastic (sarcoma) (0.554) Antineoplastic (pancreatic cancer) (0.543)	Hypolipemic (0.626)	Antithrombotic (0.689) Alzheimer’s disease treatment (0.540)
**48**	Antineoplastic (0.844) Apoptosis agonist (0.814) Chemopreventive (0.790) Antimetastatic (0.602) Antineoplastic (lymphocytic leukemia) (0.524)	Hypolipemic (0.825) Cholesterol synthesis inhibitor (0.622) Atherosclerosis treatment (0.576)	Antiviral (HIV) (0.520)
**49**	Chemopreventive (0.967) Antineoplastic (0.906) Apoptosis agonist (0.655)	Hypolipemic (0.646)	
**50**	Chemopreventive (0.936) Antineoplastic (0.895) Apoptosis agonist (0.722) Anticarcinogenic (0.604) Antineoplastic (genitourinary cancer) (0.555) Antimetastatic (0.555)	Hypolipemic (0.782)	Diabetic neuropathy treatment (0.696) Antidiabetic (0.610)
**51**	Antineoplastic (0.848) Apoptosis agonist (0.767) Chemopreventive (0.607) Antimetastatic (0.587)	Hypolipemic (0.847)	Antiprotozoal (Plasmodium) (0.629)
**52**	Antineoplastic (0.820) Chemopreventive (0.735) Cytoprotectant (0.629) Apoptosis agonist (0.598)	Anti-hypercholesterolemic (0.614) Atherosclerosis treatment (0.589) Cholesterol synthesis inhibitor (0.581)	

* Only activities with Pa > 0.5 are shown.

**Table 4:** Biological activities of carbon-bridged steroids.

No.	Antitumor & Related Activity, (Pa) *	Lipid Metabolism Regulators, (Pa) *	Additional Predicted Activity, (Pa) *
**53**	Apoptosis agonist (0.768) Antineoplastic (0.759) Chemopreventive (0.574) Antimetastatic (0.514) Antineoplastic (pancreatic cancer) (0.509)		Antiprotozoal (Plasmodium) (0.755)
**54**	Apoptosis agonist (0.778) Antineoplastic (0.770) Chemopreventive (0.634) Antineoplastic (pancreatic cancer) (0.562) Antineoplastic (sarcoma) (0.555) Antimetastatic (0.547)	Hypolipemic (0.506)	Antiprotozoal (Plasmodium) (0.724)
**55**	Antineoplastic (0.881) Apoptosis agonist (0.692) Antimetastatic (0.602)	Hypolipemic (0.775)	Cardiotonic (0.691)
**56**	Antineoplastic (0.752) Apoptosis agonist (0.698) Chemopreventive (0.619)		
**57**	Antineoplastic (0.752) Apoptosis agonist (0.698)		
**58**	Antineoplastic (0.825) Apoptosis agonist (0.690)		
**59**	Antineoplastic (0.881) Apoptosis agonist (0.728) Chemopreventive (0.709) Antineoplastic (genitourinary cancer) (0.594) Antimetastatic (0.546) Antineoplastic (sarcoma) (0.532) Antineoplastic (pancreatic cancer) (0.503)	Hypolipemic (0.805)	
**60**	Antineoplastic (0.804) Chemopreventive (0.700) Apoptosis agonist (0.669) Antineoplastic (sarcoma) (0.521) Antineoplastic (renal cancer) (0.512)	Hypolipemic (0.693) Lipid metabolism regulator (0.525)	Alzheimer’s disease treatment (0.571)
**61**	Antineoplastic (0.888) Chemopreventive (0.864) Anticarcinogenic (0.569) Antimetastatic (0.559)	Hypolipemic (0.827)	
**62**	Antineoplastic (0.869) Chemopreventive (0.862) Antimetastatic (0.560) Antineoplastic (sarcoma) (0.503)	Hypolipemic (0.815) Lipid metabolism regulator (0.520)	Antithrombotic (0.608)
**63**	Antineoplastic (0.811) Chemopreventive (0.790) Apoptosis agonist (0.774) Antineoplastic (pancreatic cancer) (0.551) Antimetastatic (0.518)	Hypolipemic (0.503)	Genital warts treatment (0.759)
**64**	Antineoplastic (0.837) Apoptosis agonist (0.803) Chemopreventive (0.748) Antineoplastic (myeloid leukemia) (0.704)	Hypolipemic (0.708) Lipid metabolism regulator (0.501)	Immunosuppressant (0.632)
**65**	Chemopreventive (0.895) Antineoplastic (0.875) Antineoplastic (myeloid leukemia) (0.677)	Hypolipemic (0.733)	

* Only activities with Pa > 0.5 are shown.

**Table 5:** Biological activities of carbon-bridged steroids.

No.	Antitumor & Related Activity, (Pa) *	Lipid Metabolism Regulators, (Pa) *	Additional Predicted Activity, (Pa) *
**66**	Chemopreventive (0.950) Antineoplastic (0.846) Proliferative diseases treatment (0.745) Anticarcinogenic (0.743) Apoptosis agonist (0.701) Antimetastatic (0.570) Antineoplastic (myeloid leukemia) (0.557) Antineoplastic (pancreatic cancer) (0.505)	Anti-hypercholesterolemic (0.769) Hypolipemic (0.752) Lipid metabolism regulator (0.730) Atherosclerosis treatment (0.532)	Hepatoprotectant (0.912)
**67**	Chemopreventive (0.948) Antineoplastic (0.861) Anticarcinogenic (0.757) Apoptosis agonist (0.740) Proliferative diseases treatment (0.712) Antimetastatic (0.576) Antineoplastic (myeloid leukemia) (0.550) Antineoplastic (lymphocytic leukemia) (0.520)	Hypolipemic (0.744) Anti-hypercholesterolemic (0.650) Lipid metabolism regulator (0.649)	Hepatoprotectant (0.903)
**68**	Chemopreventive (0.954) Antineoplastic (0.869) Apoptosis agonist (0.803) Anticarcinogenic (0.706)	Hypolipemic (0.773) Lipid metabolism regulator (0.758) Anti-hypercholesterolemic (0.751)	Hepatoprotectant (0.866)
**69**	Chemopreventive (0.943) Antineoplastic (0.835) Proliferative diseases treatment (0.719) Apoptosis agonist (0.690) Anticarcinogenic (0.656) Antineoplastic (pancreatic cancer) (0.549) Antimetastatic (0.544) Antineoplastic (sarcoma) (0.505)	Anti-hypercholesterolemic (0.798) Hypolipemic (0.675) Lipid metabolism regulator (0.513)	Hepatoprotectant (0.834)
**70**	Chemopreventive (0.958) Antineoplastic (0.859) Apoptosis agonist (0.713) Anticarcinogenic (0.634) Proliferative diseases treatment (0.597) Antimetastatic (0.562) Antineoplastic (sarcoma) (0.535) Antineoplastic (myeloid leukemia) (0.531)	Hypolipemic (0.754) Anti-hypercholesterolemic (0.606) Lipid metabolism regulator (0.511)	Anti-eczematic (0.955) Anti-psoriatic (0.592)
**71**	Chemopreventive (0.974) Antineoplastic (0.844) Anticarcinogenic (0.782) Proliferative diseases treatment (0.718) Antimetastatic (0.567) Antineoplastic (myeloid leukemia) (0.560) Antineoplastic (lymphocytic leukemia) (0.540)	Hypolipemic (0.730) Lipid metabolism regulator (0.599) Anti-hypercholesterolemic (0.519)	Respiratory analeptic (0.894)
**72**	Chemopreventive (0.808) Antineoplastic (0.782) Apoptosis agonist (0.683)	Lipid metabolism regulator (0.662) Hypolipemic (0.652)	
**73**	Antineoplastic (0.789) Chemopreventive (0.787) Apoptosis agonist (0.761) Antimetastatic (0.576) Proliferative diseases treatment (0.568) Antineoplastic (myeloid leukemia) (0.552) Cytoprotectant (0.509) Anticarcinogenic (0.503)	Lipid metabolism regulator (0.843) Hypolipemic (0.798) Cholesterol synthesis inhibitor (0.635) Anti-hypercholesterolemic (0.628)	Antithrombotic (0.638)
**74**	Antineoplastic (0.790) Apoptosis agonist (0.736) Antineoplastic (liver cancer) (0.640)	Hypolipemic (0.597)	Genital warts treatment (0.831)
**75**	Antineoplastic (0.764) Chemopreventive (0.677) Antineoplastic (liver cancer) (0.571) Apoptosis agonist (0.531)	Hypolipemic (0.679)	Genital warts treatment (0.630)
**76**	Antineoplastic (0.688)	Hypolipemic (0.553)	Genital warts treatment (0.635)
**77**	Antineoplastic (0.867) Apoptosis agonist (0.820) Antineoplastic (liver cancer) (0.561)	Hypolipemic (0.590)	Genital warts treatment (0.635)

* Only activities with Pa > 0.5 are shown.

**Table 6:** Biological activities of carbon-bridged steroids.

No.	Antitumor & Related Activity, (Pa) *	Lipid Metabolism Regulators, (Pa) *	Additional Predicted Activity, (Pa) *
**78**	Antineoplastic (0.820)		Genital warts treatment (0.780)
**79**	Antineoplastic (0.841)		Antimitotic (0.642)
**80**	Antineoplastic (0.820)		Genital warts treatment (0.780)
**81**	Antineoplastic (0.841)		Prostate disorders treatment (0.650)
**82**	Antineoplastic (0.831)		Genital warts treatment (0.854)
**83**	Antineoplastic (0.866)		Genital warts treatment (0.707)
**84**	Antineoplastic (0.759) Chemopreventive (0.711) Apoptosis agonist (0.644) Cytoprotectant (0.631) Antimetastatic (0.587)	Hypolipemic (0.764) Atherosclerosis treatment (0.600) Cholesterol synthesis inhibitor (0.525) Lipid metabolism regulator (0.521) Anti-hypercholesterolemic (0.519)	
**85**	Antineoplastic (0.801) Chemopreventive (0.780) Apoptosis agonist (0.673) Cytoprotectant (0.621) Antimetastatic (0.597)	Hypolipemic (0.765) Cholesterol synthesis inhibitor (0.577) Lipid metabolism regulator (0.500)	Immunosuppressant (0.727)
**86**	Antineoplastic (0.773) Apoptosis agonist (0.687) Chemopreventive (0.609) Cytoprotectant (0.583)	Cholesterol synthesis inhibitor (0.556) Hypolipemic (0.511)	Anti-ischemic, cerebral (0.973)
**87**	Antineoplastic (0.825) Antineoplastic (myeloid leukemia) (0.645) Apoptosis agonist (0.573) Antineoplastic (carcinoma) (0.504)		Alzheimer’s disease treatment (0.824) Neurodegenerative diseases treatment (0.809) Psychotropic (0.700)
**88**	Antineoplastic (0.889) Apoptosis agonist (0.580) Antimetastatic (0.515)	Hypolipemic (0.508)	Hepatic disorders treatment (0.931)
**89**	Antineoplastic (0.870) Apoptosis agonist (0.759)		Hepatic disorders treatment (0.952) Hepatoprotectant (0.514)

* Only activities with Pa > 0.5 are shown.

**Table 7:** Biological activities of carbon-bridged steroids.

No.	Antitumor & Related Activity, (Pa) *	Lipid Metabolism Regulators, (Pa) *	Additional Predicted Activity, (Pa) *
**90**	Antineoplastic (0.715)		Immunosuppressant (0.770) Cardiotonic (0.726)
**91**	Antineoplastic (0.744)		Immunosuppressant (0.735) Cardiotonic (0.688)
**92**	Antineoplastic (0.901) Apoptosis agonist (0.818) Chemopreventive (0.732) Cytostatic (0.606) Antimetastatic (0.581) Anticarcinogenic (0.546) Antineoplastic (breast cancer) (0.539)	Anti-hypercholesterolemic (0.625) Hypolipemic (0.617)	Respiratory analeptic (0.902) Antidepressant (0.776)
**93**	Antineoplastic (0.839) Proliferative diseases treatment (0.804) Chemopreventive (0.792) Anticarcinogenic (0.722) Apoptosis agonist (0.701) Antineoplastic (sarcoma) (0.567) Antimetastatic (0.503)	Lipoprotein disorders treatment (0.800) Anti-hypercholesterolemic (0.677)	Antidiabetic (0.902) Spasmolytic (0.705) Cardiotonic (0.682)
**94**	Antineoplastic (0.877) Chemopreventive (0.709) Apoptosis agonist (0.707) Antineoplastic (sarcoma) (0.673) Proliferative diseases treatment (0.630) Antineoplastic (lymphocytic leukemia) (0.560) Prostate disorders treatment (0.557) Cytostatic (0.557) Anticarcinogenic (0.556) Antineoplastic (pancreatic cancer) (0.538) Antineoplastic (breast cancer) (0.522) Antimetastatic (0.505)	Anti-hypercholesterolemic (0.862) Lipid metabolism regulator (0.549) Hypolipemic (0.532)	Respiratory analeptic (0.950)
**95**	Antineoplastic (0.856) Chemopreventive (0.701) Antineoplastic (sarcoma) (0.688) Proliferative diseases treatment (0.640) Apoptosis agonist (0.615) Anticarcinogenic (0.588) Cytostatic (0.584) Antineoplastic (lymphocytic leukemia) (0.569) Antineoplastic (pancreatic cancer) (0.534) Antineoplastic (renal cancer) (0.531) Antimetastatic (0.512)	Anti-hypercholesterolemic (0.806) Lipid metabolism regulator (0.539)	Respiratory analeptic (0.953) Hepatoprotectant (0.901)
**96**	Antineoplastic (0.937) Apoptosis agonist (0.827) Chemopreventive (0.757) Anticarcinogenic (0.741) Proliferative diseases treatment (0.718) Antineoplastic (sarcoma) (0.673) Antineoplastic (lymphocytic leukemia) (0.587) Antimetastatic (0.540) Antineoplastic (breast cancer) (0.525) Antineoplastic (pancreatic cancer) (0.524) Antineoplastic (renal cancer) (0.521)	Anti-hypercholesterolemic (0.863) Lipid metabolism regulator (0.555)	Respiratory analeptic (0.952)
**97**	Antineoplastic (0.801) Antineoplastic (breast cancer) (0.603) Apoptosis agonist (0.589)		Antidepressant (0.946) Mood disorders treatment (0.944) Psychotropic (0.922)
**98**	Antineoplastic (0.763) Antineoplastic (genitourinary cancer) (0.537) Antimetastatic (0.514)		Antiprotozoal (0.955) Antiprotozoal (Plasmodium) (0.950)
**99**	Antineoplastic (0.875) Chemopreventive (0.648) Antineoplastic (sarcoma) (0.633) Apoptosis agonist (0.630) Proliferative diseases treatment (0.566) Antimetastatic (0.523) Antineoplastic (lymphocytic leukemia) (0.512) Anticarcinogenic (0.506)		Anti-ischemic, cerebral (0.770) Immunosuppressant (0.747)
**100**	Antineoplastic (0.875) Chemopreventive (0.648) Antineoplastic (sarcoma) (0.633) Apoptosis agonist (0.630) Proliferative diseases treatment (0.566) Antimetastatic (0.523) Anticarcinogenic (0.506)		Anti-ischemic, cerebral (0.770) Immunosuppressant (0.747)
**101**	Antineoplastic (0.869) Anticarcinogenic (0.823) Proliferative diseases treatment (0.781) Chemopreventive (0.717) Apoptosis agonist (0.667) Antineoplastic (sarcoma) (0.560) Antimetastatic (0.516)		Anti-ischemic, cerebral (0.702)
**102**	Cytoprotectant (0.758) Antineoplastic (0.720) Chemopreventive (0.591) Apoptosis agonist (0.564)	Hypolipemic (0.679) Anti-hypercholesterolemic (0.599) Atherosclerosis treatment (0.541) Cholesterol synthesis inhibitor (0.533)	Choleretic (0.733)

* Only activities with Pa > 0.5 are shown.

**Table 8:** Biological activities of sterols and triterpenoids with cyclopropane ring in the side chain.

No.	Antitumor & Related Activity, (Pa) *	Lipid Metabolism Regulators, (Pa) *	Additional Predicted Activity, (Pa) *
**103**	Antineoplastic (0.911) Apoptosis agonist (0.677) Chemopreventive (0.658) Cytoprotectant (0.630) Antineoplastic (sarcoma) (0.558)	Anti-hypercholesterolemic (0.791) Hypolipemic (0.789)	Choleretic (0.885)
**104**	Antineoplastic (0.822) Proliferative diseases treatment (0.668) Cytoprotectant (0.635) Chemopreventive (0.557) Apoptosis agonist (0.536) Antineoplastic (sarcoma) (0.530) Antimetastatic (0.518)	Anti-hypercholesterolemic (0.862) Hypolipemic (0.757) Cholesterol synthesis inhibitor (0.517)	Anti-ischemic, cerebral (0.952) Choleretic (0.935)
**105**	Antineoplastic (0.934) Proliferative diseases treatment (0.644) Prostate cancer treatment (0.585) Antineoplastic (sarcoma) (0.575) Cytoprotectant (0.544) Antineoplastic (renal cancer) (0.520) Apoptosis agonist (0.517)	Anti-hypercholesterolemic (0.828) Hypolipemic (0.709)	Choleretic (0.879) Anti-ischemic, cerebral (0.674)
**106**	Antineoplastic (0.839) Chemopreventive (0.697) Cytoprotectant (0.670) Proliferative diseases treatment (0.642) Apoptosis agonist (0.607) Prostatic (benign) hyperplasia treatment (0.520) Antimetastatic (0.515) Antineoplastic (renal cancer) (0.513)	Anti-hypercholesterolemic (0.850) Hypolipemic (0.728)	Choleretic (0.910)
**107**	Antineoplastic (0.849) Chemopreventive (0.789) Proliferative diseases treatment (0.785) Apoptosis agonist (0.750) Cytoprotectant (0.717) Anticarcinogenic (0.658) Prostate cancer treatment (0.601) Antimetastatic (0.584) Antineoplastic (sarcoma) (0.578) Antineoplastic (pancreatic cancer) (0.547)	Anti-hypercholesterolemic (0.964) Hypolipemic (0.849) Anti-hyperlipoproteinemic (0.801) Cholesterol synthesis inhibitor (0.671) Atherosclerosis treatment (0.610)	Respiratory analeptic (0.964) Choleretic (0.856)
**108**	Antineoplastic (0.861) Antimetastatic (0.552)		Angiogenesis inhibitor (0.928)
**109**	Antineoplastic (0.821) Chemopreventive (0.743) Prostatic (benign) hyperplasia treatment (0.663) Cytoprotectant (0.660) Proliferative diseases treatment (0.648) Apoptosis agonist (0.594) Antimetastatic (0.550) Prostate cancer treatment (0.538)	Anti-hypercholesterolemic (0.923) Hypolipemic (0.732) Atherosclerosis treatment (0.643) Cholesterol synthesis inhibitor (0.640)	Respiratory analeptic (0.844) Anesthetic general (0.834)
**110**	Antineoplastic (0.821) Chemopreventive (0.743) Prostatic (benign) hyperplasia treatment (0.663) Cytoprotectant (0.660) Proliferative diseases treatment (0.648) Apoptosis agonist (0.594) Antimetastatic (0.550) Prostate cancer treatment (0.538)	Anti-hypercholesterolemic (0.923) Hypolipemic (0.732) Atherosclerosis treatment (0.643) Cholesterol synthesis inhibitor (0.640)	
**111**	Antineoplastic (0.898) Apoptosis agonist (0.586) Cytoprotectant (0.553) Antineoplastic (sarcoma) (0.516)	Hypolipemic (0.778) Anti-hypercholesterolemic (0.520)	Choleretic (0.711) Antiprotozoal (Plasmodium) (0.640)
**112**	Antineoplastic (0.922) Prostate disorders treatment (0.553) Proliferative diseases treatment (0.545) Antineoplastic (sarcoma) (0.536)	Hypolipemic (0.692) Anti-hypercholesterolemic (0.578)	Choleretic (0.707)
**113**	Antineoplastic (0.845) Chemopreventive (0.734) Cytoprotectant (0.730) Proliferative diseases treatment (0.700) Antimetastatic (0.634) Anticarcinogenic (0.607) Prostate cancer treatment (0.533)	Anti-hypercholesterolemic (0.909) Hypolipemic (0.872) Atherosclerosis treatment (0.639) Cholesterol synthesis inhibitor (0.584)	Choleretic (0.962)
**114**	Antineoplastic (0.832) Cytoprotectant (0.668) Proliferative diseases treatment (0.659) Chemopreventive (0.611) Antineoplastic (sarcoma) (0.555) Prostatic (benign) hyperplasia treatment (0.500)	Anti-hypercholesterolemic (0.865) Hypolipemic (0.743) Atherosclerosis treatment (0.553) Cholesterol synthesis inhibitor (0.514)	Choleretic (0.934) Respiratory analeptic (0.897)
**115**	Antineoplastic (0.858) Cytoprotectant (0.699) Antineoplastic (sarcoma) (0.685) Antimetastatic (0.591) Antineoplastic (renal cancer) (0.585) Prostate disorders treatment (0.578) Proliferative diseases treatment (0.554) Apoptosis agonist (0.549) Antineoplastic (pancreatic cancer) (0.531) Chemopreventive (0.522) Antineoplastic (genitourinary cancer) (0.506)	Hypolipemic (0.713)	Immunosuppressant (0.780)
**116**	Antineoplastic (0.682) Prostate disorders treatment (0.670) Apoptosis agonist (0.613) Chemopreventive (0.604) Cytoprotectant (0.566) Prostatic (benign) hyperplasia treatment (0.532) Antimetastatic (0.527)	Anti-hypercholesterolemic (0.836) Cholesterol synthesis inhibitor (0.587) Hypolipemic (0.563)	
**117**	Antineoplastic (0.706) Prostate disorders treatment (0.630) Cytoprotectant (0.600) Antimetastatic (0.555) Prostatic (benign) hyperplasia treatment (0.510)	Hypolipemic (0.587) Cholesterol synthesis inhibitor (0.509)	Immunosuppressant (0.720)

* Only activities with Pa > 0.5 are shown.

**Table 9:** Biological activities of sterols and triterpenoids with cyclopropane ring in the side chain.

No.	Antitumor & Related Activity, (Pa) *	Lipid Metabolism Regulators, (Pa) *	Additional Predicted Activity, (Pa) *
**118**	Chemopreventive (0.963) Proliferative diseases treatment (0.931) Antineoplastic (0.885) Anticarcinogenic (0.861) Apoptosis agonist (0.790) Antineoplastic (sarcoma) (0.624) Antimetastatic (0.569) Antineoplastic (liver cancer) (0.529) Antineoplastic (lymphocytic leukemia) (0.516) Antineoplastic (pancreatic cancer) (0.502)	Anti-hypercholesterolemic (0.953) Hypolipemic (0.758) Lipid metabolism regulator (0.674) Atherosclerosis treatment (0.513)	Respiratory analeptic (0.982) Hepatoprotectant (0.979)
**119**	Chemopreventive (0.960) Proliferative diseases treatment (0.921) Antineoplastic (0.904) Anticarcinogenic (0.851) Apoptosis agonist (0.824) Antineoplastic (sarcoma) (0.633) Antimetastatic (0.569) Prostate disorders treatment (0.548) Antineoplastic (liver cancer) (0.543)	Anti-hypercholesterolemic (0.939) Hypolipemic (0.746) Lipid metabolism regulator (0.599)	Respiratory analeptic (0.987) Hepatoprotectant (0.984) Antiprotozoal (Leishmania) (0.880)
**120**	Apoptosis agonist (0.975) Chemopreventive (0.916) Antineoplastic (0.845) Prostate disorders treatment (0.615) Cytoprotectant (0.611) Antimetastatic (0.543)	Atherosclerosis treatment (0.731) Hypolipemic (0.632)	Antiprotozoal (Plasmodium) (0.768)
**121**	Antineoplastic (0.845) Chemopreventive (0.832) Apoptosis agonist (0.822) Proliferative diseases treatment (0.818) Prostate cancer treatment (0.584) Antimetastatic (0.537) Antineoplastic (sarcoma) (0.531)	Anti-hypercholesterolemic (0.969) Hypolipemic (0.810) Lipid metabolism regulator (0.716) Cholesterol synthesis inhibitor (0.707) Atherosclerosis treatment (0.586)	Wound healing agent (0.916) Respiratory analeptic (0.902)
**122**	Antineoplastic (0.818) Chemopreventive (0.742) Apoptosis agonist (0.690) Prostatic (benign) hyperplasia treatment (0.660) Cytoprotectant (0.642) Proliferative diseases treatment (0.622) Antimetastatic (0.556) Prostate cancer treatment (0.541)	Anti-hypercholesterolemic (0.903) Hypolipemic (0.709) Atherosclerosis treatment (0.613) Cholesterol synthesis inhibitor (0.595)	Anesthetic general (0.884) Respiratory analeptic (0.876)
**123**	Chemopreventive (0.857) Antineoplastic (0.850) Apoptosis agonist (0.759) Cytoprotectant (0.723) Prostatic (benign) hyperplasia treatment (0.685) Proliferative diseases treatment (0.671) Antimetastatic (0.568) Prostate cancer treatment (0.557) Antineoplastic (pancreatic cancer) (0.530) Anticarcinogenic (0.517) Antineoplastic (breast cancer) (0.516)	Anti-hypercholesterolemic (0.961) Hypolipemic (0.755) Atherosclerosis treatment (0.690) Cholesterol synthesis inhibitor (0.652) Anti-hyperlipoproteinemic (0.607) Lipid metabolism regulator (0.572)	Respiratory analeptic (0.901)
**124**	Antineoplastic (0.753) Apoptosis agonist (0.677) Prostate disorders treatment (0.584)		
**125**	Antineoplastic (0.791) Prostate disorders treatment (0.613) Proliferative diseases treatment (0.556)	Anti-hypercholesterolemic (0.704) Hypolipemic (0.556) Cholesterol synthesis inhibitor (0.530)	Anti-inflammatory (0.833)
**126**	Antineoplastic (0.697)	Anti-hypercholesterolemic (0.555) Cholesterol synthesis inhibitor (0.504)	
**127**	Apoptosis agonist (0.756) Antineoplastic (0.660)		Antiprotozoal (Plasmodium) (0.687)
**128**	Antineoplastic (0.731) Apoptosis agonist (0.599)	Anti-hypercholesterolemic (0.571) Hypolipemic (0.546)	
**129**	Antineoplastic (0.824) Chemopreventive (0.726) Proliferative diseases treatment (0.657) Prostatic (benign) hyperplasia treatment (0.656) Cytoprotectant (0.654) Apoptosis agonist (0.637) Antimetastatic (0.539) Prostate cancer treatment (0.538) Antineoplastic (sarcoma) (0.537) Antineoplastic (breast cancer) (0.507)	Anti-hypercholesterolemic (0.935) Hypolipemic (0.731) Anti-hyperlipoproteinemic (0.689) Cholesterol synthesis inhibitor (0.600)	Anti-eczematic (0.961) Respiratory analeptic (0.904)
**130**	Antineoplastic (0.813) Chemopreventive (0.717) Proliferative diseases treatment (0.695) Cytoprotectant (0.670) Prostatic (benign) hyperplasia treatment (0.649) Antineoplastic (sarcoma) (0.628) Apoptosis agonist (0.608) Prostate cancer treatment (0.559) Anticarcinogenic (0.556) Antineoplastic (pancreatic cancer) (0.550) Antineoplastic (breast cancer) (0.528) Antimetastatic (0.524) Antineoplastic (renal cancer) (0.514)	Anti-hypercholesterolemic (0.908) Hypolipemic (0.726) Cholesterol synthesis inhibitor (0.589) Anti-hyperlipoproteinemic (0.587)	Anti-eczematic (0.960) Respiratory analeptic (0.905)

* Only activities with Pa > 0.5 are shown.

**Table 10:** Biological activities of cyclopropane-containing steroids and triterpenoids.

No.	Antitumor & Related Activity, (Pa) *	Lipid Metabolism Regulators, (Pa) *	Additional Predicted Activity, (Pa) *
**131**	Antineoplastic (0.780) Apoptosis agonist (0.559) Antimetastatic (0.549) Antineoplastic (myeloid leukemia) (0.537)	Hypolipemic (0.577) Lipid metabolism regulator (0.567)	
**132**	Antineoplastic (0.780) Apoptosis agonist (0.559) Antimetastatic (0.549) Antineoplastic (myeloid leukemia) (0.537)	Hypolipemic (0.577) Lipid metabolism regulator (0.567)	
**133**	Antineoplastic (0.769) Apoptosis agonist (0.576) Antimetastatic (0.547)	Hypolipemic (0.660) Lipid metabolism regulator (0.604)	
**134**	Antineoplastic (0.769) Apoptosis agonist (0.576) Antimetastatic (0.547)	Hypolipemic (0.660) Lipid metabolism regulator (0.604)	
**135**	Antineoplastic (0.811) Apoptosis agonist (0.639) Chemopreventive (0.560) Antineoplastic (myeloid leukemia) (0.545) Antimetastatic (0.562)	Hypolipemic (0.629) Lipid metabolism regulator (0.544)	
**136**	Antineoplastic (0.795) Apoptosis agonist (0.625) Prostate disorders treatment (0.605) Antineoplastic (sarcoma) (0.574) Chemopreventive (0.573) Antineoplastic (myeloid leukemia) (0.538)	Hypolipemic (0.597) Lipid metabolism regulator (0.537)	Hepatoprotectant (0.791)
**137**	Antineoplastic (0.758) Chemopreventive (0.661) Prostate disorders treatment (0.654) Apoptosis agonist (0.643) Cytoprotectant (0.621) Proliferative diseases treatment (0.590) Antimetastatic (0.588) Prostatic (benign) hyperplasia treatment (0.512)	Anti-hypercholesterolemic (0.895) Hypolipemic (0.707) Cholesterol synthesis inhibitor (0.549) Atherosclerosis treatment (0.533)	Anti-eczematic (0.849) Anti-psoriatic (0.691)
**138**	Antineoplastic (0.758) Chemopreventive (0.661) Prostate disorders treatment (0.654) Apoptosis agonist (0.643) Cytoprotectant (0.621) Proliferative diseases treatment (0.590) Antimetastatic (0.588)	Anti-hypercholesterolemic (0.895) Cholesterol synthesis inhibitor (0.549) Atherosclerosis treatment (0.533)	Anti-eczematic (0.849) Anti-psoriatic (0.691)
**139**	Antineoplastic (0.809) Cytoprotectant (0.681) Chemopreventive (0.670) Apoptosis agonist (0.647) Antimetastatic (0.635) Proliferative diseases treatment (0.635) Prostate disorders treatment (0.632) Antineoplastic (pancreatic cancer) (0.509)	Anti-hypercholesterolemic (0.797) Hypolipemic (0.709) Cholesterol synthesis inhibitor (0.557)	Anti-eczematic (0.921) Anti-psoriatic (0.780)
**140**	Antineoplastic (0.724) Antimetastatic (0.695) Apoptosis agonist (0.626)		
**141**	Antineoplastic (0.855) Apoptosis agonist (0.637) Antimetastatic (0.504)		
**142**	Antineoplastic (0.688) Antineoplastic (renal cancer) (0.524)		
**143**	Apoptosis agonist (0.908) Antineoplastic (0.857) Chemopreventive (0.804) Antineoplastic (liver cancer) (0.797) Proliferative diseases treatment (0.587) Prostate cancer treatment (0.507)	Hypolipemic (0.788) Atherosclerosis treatment (0.625) Cholesterol synthesis inhibitor (0.548)	Anti-eczematic (0.828)
**144**	Antineoplastic (0.812) Chemopreventive (0.619) Cytoprotectant (0.558) Antimetastatic (0.521)	Hypolipemic (0.701)	Anti-inflammatory (0.862)
**145**	Apoptosis agonist (0.870) Antineoplastic (0.824) Chemopreventive (0.647)	Hypolipemic (0.710)	Anti-inflammatory (0.801)
**146**	Chemopreventive (0.987) Antineoplastic (0.858) Anticarcinogenic (0.815) Apoptosis agonist (0.802) Proliferative diseases treatment (0.660)	Atherosclerosis treatment (0.640) Anti-hypercholesterolemic (0.635) Hypolipemic (0.511)	Hepatoprotectant (0.993) Wound healing agent (0.872)
**147**	Chemopreventive (0.980) Antineoplastic (0.852) Anticarcinogenic (0.792) Apoptosis agonist (0.787) Proliferative diseases treatment (0.631)	Atherosclerosis treatment (0.645) Anti-hypercholesterolemic (0.640)	Hepatoprotectant (0.988) Wound healing agent (0.925)
**148**	Chemopreventive (0.969) Antineoplastic (0.867) Apoptosis agonist (0.801) Anticarcinogenic (0.775) Proliferative diseases treatment (0.625)	Atherosclerosis treatment (0.663) Anti-hypercholesterolemic (0.611) Hypolipemic (0.539)	Hepatoprotectant (0.987) Wound healing agent (0.949)

* Only activities with Pa > 0.5 are shown.

**Table 11:** Biological activities of synthetic cyclopropane-containing steroids.

No.	Antitumor & Related Activity, (Pa) *	Lipid Metabolism Regulators, (Pa) *	Additional Predicted Activity, (Pa) *
**149**	Antineoplastic (0.891) Apoptosis agonist (0.665)		Antidepressant (0.954) Psychotropic (0.919)
**150**	Antineoplastic (0.871) Apoptosis agonist (0.814) Prostate disorders treatment (0.699) Cytoprotectant (0.670)		Antidepressant (0.961) Psychotropic (0.953)
**151**	Antineoplastic (0.845)	Atherosclerosis treatment (0.600)	Cardiovascular analeptic (0.828)
**152**	Antineoplastic (0.827) Prostate disorders treatment (0.723) Prostatic (benign) hyperplasia treatment (0.619)	Anti-hypercholesterolemic (0.642)	Anti-seborrheic (0.905)
**153**	Antineoplastic (0.877) Apoptosis agonist (0.611)		Anti-seborrheic (0.849)
**154**	Antineoplastic (0.864) Prostate disorders treatment (0.731) Prostatic (benign) hyperplasia treatment (0.652) Prostate cancer treatment (0.564)		Anti-seborrheic (0.844)
**155**	Antineoplastic (0.905) Prostate disorders treatment (0.742) Prostatic (benign) hyperplasia treatment (0.621)		Anti-seborrheic (0.823)
**156**	Antineoplastic (0.791) Cytoprotectant (0.713) Proliferative diseases treatment (0.662)	Anti-hypercholesterolemic (0.881) Hypolipemic (0.735) Cholesterol synthesis inhibitor (0.641)	Anti-eczematic (0.850)
**157**	Antineoplastic (0.744) Prostate disorders treatment (0.677) Cytoprotectant (0.653) Prostatic (benign) hyperplasia treatment (0.589)	Anti-hypercholesterolemic (0.873) Hypolipemic (0.789) Cholesterol synthesis inhibitor (0.619)	Respiratory analeptic (0.898)
**158**	Antineoplastic (0.851) Apoptosis agonist (0.634) Prostate cancer treatment (0.613) Prostatic (benign) hyperplasia treatment (0.592)	Aldosterone antagonist (0.842) Anti-hyperaldosteronism (0.842)	Diuretic (0.973) Mineralocorticoid antagonist (0.956) Antihypertensive (0.802)
**159**	Antineoplastic (0.841) Prostatic (benign) hyperplasia treatment (0.636) Cytoprotectant (0.620)		Anti-seborrheic (0.892)
**160**	Antineoplastic (0.749) Prostate disorders treatment (0.737) Prostatic (benign) hyperplasia treatment (0.603)	Anti-hypercholesterolemic (0.580)	Respiratory analeptic (0.765) Cardiovascular analeptic (0.745)
**161**	Antineoplastic (0.792) Prostate disorders treatment (0.742) Prostatic (benign) hyperplasia treatment (0.657)	Anti-hypercholesterolemic (0.909) Hypolipemic (0.602)	
**162**	Antineoplastic (0.849) Prostate disorders treatment (0.733) Prostatic (benign) hyperplasia treatment (0.665)	Anti-hypercholesterolemic (0.666)	Erythropoiesis stimulant (0.816)
**163**	Antineoplastic (0.849) Apoptosis agonist (0.750) Prostate disorders treatment (0.744) Prostate cancer treatment (0.601)	Anti-hypercholesterolemic (0.964) Atherosclerosis treatment (0.610)	Respiratory analeptic (0.964) Anesthetic general (0.898)
**164**	Antineoplastic (0.714) Cytoprotectant (0.710) Prostate disorders treatment (0.619)	Hypolipemic (0.689) Anti-hypercholesterolemic (0.625)	Respiratory analeptic (0.863) Erythropoiesis stimulant (0.784)

* Only activities with Pa > 0.5 are shown.

**Table 12:** Biological activities of cyclobutane-containing steroids and triterpenoids.

No.	Antitumor & Related Activity, (Pa) *	Lipid Metabolism Regulators, (Pa) *	Additional Predicted Activity, (Pa) *
**165**	Antineoplastic (0.754) Chemopreventive (0.703) Cytoprotectant (0.609) Apoptosis agonist (0.602) Antineoplastic (pancreatic cancer) (0.532) Antimetastatic (0.523) Prostate disorders treatment (0.505)	Hypolipemic (0.541)	Anti-eczematic (0.905) Anti-psoriatic (0.650)
**166**	Antineoplastic (0.730) Chemopreventive (0.693) Cytoprotectant (0.608) Apoptosis agonist (0.572) Antimetastatic (0.517) Antineoplastic (pancreatic cancer) (0.512)	Hypolipemic (0.571)	Anti-eczematic (0.899) Anti-psoriatic (0.650)
**167**	Antineoplastic (0.744) Chemopreventive (0.706) Cytoprotectant (0.627) Apoptosis agonist (0.526) Antimetastatic (0.510) Antineoplastic (pancreatic cancer) (0.503)	Hypolipemic (0.515)	Anti-eczematic (0.895) Anti-psoriatic (0.656)
**168**	Antineoplastic (0.796) Apoptosis agonist (0.667) Cytoprotectant (0.621) Chemopreventive (0.599)	Hypolipemic (0.588) Atherosclerosis treatment (0.528)	
**169**	Antineoplastic (0.768) Chemopreventive (0.628) Apoptosis agonist (0.574)	Hypolipemic (0.638)	
**170**	Antineoplastic (0.780) Apoptosis agonist (0.675) Cytoprotectant (0.602) Chemopreventive (0.599)	Hypolipemic (0.560)	
**171**	Antineoplastic (0.821) Apoptosis agonist (0.740) Chemopreventive (0.726) Cytoprotectant (0.707) Proliferative diseases treatment (0.553) Prostate cancer treatment (0.551) Antineoplastic (pancreatic cancer) (0.538)	Lipid metabolism regulator (0.794) Anti-hypercholesterolemic (0.738) Hypolipemic (0.709) Cholesterol synthesis inhibitor (0.574)	Anti-secretoric (0.823)
**172**	Antineoplastic (0.847) Antineoplastic (myeloid leukemia) (0.624)		
**173**	Antineoplastic (0.786) Apoptosis agonist (0.725) Antineoplastic (sarcoma) (0.643) Antimetastatic (0.580) Antineoplastic (renal cancer) (0.500)	Hypolipemic (0.543)	
**174**	Antineoplastic (0.781) Apoptosis agonist (0.722) Antineoplastic (sarcoma) (0.635) Antimetastatic (0.572)	Hypolipemic (0.534)	
**175**	Antineoplastic (0.897) Chemopreventive (0.718) Apoptosis agonist (0.658) Antimetastatic (0.649) Antineoplastic (renal cancer) (0.611) Prostate cancer treatment (0.595) Antineoplastic (pancreatic cancer) (0.547)	Hypolipemic (0.663)	
**176**	Antineoplastic (0.850) Chemopreventive (0.847) Apoptosis agonist (0.829) Cytoprotectant (0.665) Antimetastatic (0.604) Antineoplastic (pancreatic cancer) (0.539)	Hypolipemic (0.567) Cholesterol synthesis inhibitor (0.529)	Anti-inflammatory (0.902) Choleretic (0.726)
**177**	Antineoplastic (0.819) Apoptosis agonist (0.746)		Antiviral (Influenza) (0.647)
**178**	Antineoplastic (0.820) Apoptosis agonist (0.795) Chemopreventive (0.601) Cytoprotectant (0.594) Antimetastatic (0.533)	Hypolipemic (0.592)	Anti-inflammatory (0.826)
**179**	Antineoplastic (0.820) Apoptosis agonist (0.795) Chemopreventive (0.601) Cytoprotectant (0.594) Antimetastatic (0.533)	Hypolipemic (0.592)	Anti-inflammatory (0.826)
**180**	Antineoplastic (0.853) Apoptosis agonist (0.848) Chemopreventive (0.717) Cytoprotectant (0.636) Antimetastatic (0.543) Antineoplastic (myeloid leukemia) (0.523)	Hypolipemic (0.616)	Anti-inflammatory (0.757)
**181**	Antineoplastic (0.853) Apoptosis agonist (0.848) Chemopreventive (0.717) Cytoprotectant (0.636) Antimetastatic (0.543) Antineoplastic (myeloid leukemia) (0.523)	Hypolipemic (0.616)	Anti-inflammatory (0.757)
**182**	Antineoplastic (0.772) Apoptosis agonist (0.764) Cytoprotectant (0.684) Antineoplastic (multiple myeloma) (0.631) Antineoplastic (pancreatic cancer) (0.589) Antineoplastic (carcinoma) (0.571) Antineoplastic (squamous cell carcinoma) (0.571) Antimetastatic (0.565)	Hypolipemic (0.765)	Anti-inflammatory (0.855)
**183**	Antineoplastic (0.774) Apoptosis agonist (0.730) Cytoprotectant (0.597) Antineoplastic (pancreatic cancer) (0.573) Antineoplastic (multiple myeloma) (0.565) Antineoplastic (carcinoma) (0.559) Antineoplastic (squamous cell carcinoma) (0.559) Antimetastatic (0.510)	Hypolipemic (0.797) Lipid metabolism regulator (0.571)	Anti-inflammatory (0.851)

* Only activities with Pa > 0.5 are shown.

**Table 13:** Biological activities of cyclobutane-containing steroids and triterpenoids.

No.	Antitumor & Related Activity, (Pa) *	Lipid Metabolism Regulators, (Pa) *	Additional Predicted Activity, (Pa) *
**184**	Antineoplastic (0.805) Apoptosis agonist (0.787) Chemopreventive (0.603) Cytoprotectant (0.586) Antimetastatic (0.533)	Hypolipemic (0.615) Lipid metabolism regulator (0.511) Anti-hypercholesterolemic (0.503)	Anti-inflammatory (0.817) Choleretic (0.771)
**185**	Antineoplastic (0.805) Apoptosis agonist (0.787) Chemopreventive (0.603) Cytoprotectant (0.586) Antimetastatic (0.533)	Hypolipemic (0.615) Lipid metabolism regulator (0.511) Anti-hypercholesterolemic (0.503)	Anti-inflammatory (0.817) Choleretic (0.771)
**186**	Antineoplastic (0.802) Apoptosis agonist (0.782) Chemopreventive (0.636) Antimetastatic (0.547)	Hypolipemic (0.598) Anti-hypercholesterolemic (0.515)	Anti-inflammatory (0.803) Choleretic (0.706)
**187**	Antineoplastic (0.802) Apoptosis agonist (0.782) Chemopreventive (0.636) Antimetastatic (0.547)	Hypolipemic (0.598) Anti-hypercholesterolemic (0.515)	Anti-inflammatory (0.803) Choleretic (0.706)
**188**	Antineoplastic (0.866) Apoptosis agonist (0.671)		Genital warts treatment (0.744)
**189**	Antineoplastic (0.863) Apoptosis agonist (0.584)		Genital warts treatment (0.736)
**190**	Antineoplastic (0.846) Apoptosis agonist (0.553)		Genital warts treatment (0.745)
**191**	Antineoplastic (0.850) Apoptosis agonist (0.577)		Genital warts treatment (0.675)
**192**	Antineoplastic (0.847)		Genital warts treatment (0.671)
**193**	Antineoplastic (0.844)		Genital warts treatment (0.664)
**194**	Apoptosis agonist (0.684)		Genital warts treatment (0.707)
**195**	Antineoplastic (0.845)		Genital warts treatment (0.682)
**196**	Antineoplastic (0.863)		Genital warts treatment (0.736)

* Only activities with Pa > 0.5 are shown.

**Table 14:** Bioactive natural and synthetic cyclobutane-containing steroids and triterpenoids.

No.	Antitumor & Related Activity, (Pa) *	Lipid Metabolism Regulators, (Pa) *	Additional Predicted Activity, (Pa) *
**197**	Antineoplastic (0.929) Prostatic (benign) hyperplasia treatment (0.663) Prostate cancer treatment (0.570)	Anti-hypercholesterolemic (0.696) Immunosuppressant (0.672) Lipid metabolism regulator (0.604)	Anti-seborrheic (0.907)
**198**	Antineoplastic (0.784) Apoptosis agonist (0.627) Cytoprotectant (0.558) Chemopreventive (0.542)	Anti-hypercholesterolemic (0.724) Hypolipemic (0.645)	Anesthetic (0.921) Neuroprotector (0.880) Psychostimulant (0.675)
**199**	Antineoplastic (0.889) Proliferative diseases treatment (0.676) Prostate disorders treatment (0.628) Cytoprotectant (0.627) Antimetastatic (0.617) Apoptosis agonist (0.614) Chemopreventive (0.606) Antineoplastic (pancreatic cancer) (0.530)	Anti-hypercholesterolemic (0.902) Hypolipemic (0.721) Cholesterol synthesis inhibitor (0.534)	Anti-eczematic (0.911) Choleretic (0.839)
**200**	Antineoplastic (0.801) Apoptosis agonist (0.706) Proliferative diseases treatment (0.667) Chemopreventive (0.665) Cytoprotectant (0.616) Antimetastatic (0.598) Prostatic (benign) hyperplasia treatment (0.528)	Anti-hypercholesterolemic (0.932) Hypolipemic (0.695) Cholesterol synthesis inhibitor (0.588)	Anti-eczematic (0.871) Choleretic (0.791)
**201**	Antineoplastic (0.865) Cytoprotectant (0.669) Antineoplastic (breast cancer) (0.662) Antineoplastic (renal cancer) (0.602) Apoptosis agonist (0.602) Antineoplastic (sarcoma) (0.588) Prostate cancer treatment (0.557) Proliferative diseases treatment (0.548)	Anti-hypercholesterolemic (0.740) Lipid metabolism regulator (0.643) Hypolipemic (0.613)	Anti-seborrheic (0.946) Anti-eczematic (0.723)
**202**	Antineoplastic (0.757) Prostate disorders treatment (0.652) Antineoplastic (breast cancer) (0.637) Apoptosis agonist (0.541)		Anti-seborrheic (0.841) Cardiotonic (0.654) Psychosexual dysfunction treatment (0.575)
**203**	Antineoplastic (0.719) Antineoplastic (breast cancer) (0.540)	Hypolipemic (0.810)	Anti-seborrheic (0.818) Cardiotonic (0.691)
**204**	Antineoplastic (0.872) Antineoplastic (sarcoma) (0.683) Antineoplastic (breast cancer) (0.625) Apoptosis agonist (0.621) Antineoplastic (renal cancer) (0.605) Prostate cancer treatment (0.548) Antineoplastic (pancreatic cancer) (0.546)	Anti-hypercholesterolemic (0.616) Lipid metabolism regulator (0.565) Hypolipemic (0.546)	Anti-seborrheic (0.917) Anti-secretoric (0.908)
**205**	Antineoplastic (0.778) Prostate disorders treatment (0.737) Prostatic (benign) hyperplasia treatment (0.617) Cytoprotectant (0.616) Antimetastatic (0.571) Proliferative diseases treatment (0.527)	Anti-hypercholesterolemic (0.638) Hypolipemic (0.542) Cholesterol synthesis inhibitor (0.535)	Anti-eczematic (0.831) Anti-osteoporotic (0.799)
**206**	Antineoplastic (0.908) Prostate disorders treatment (0.703) Antineoplastic (breast cancer) (0.635) Antineoplastic (renal cancer) (0.596) Antineoplastic (sarcoma) (0.567) Prostate cancer treatment (0.553) Apoptosis agonist (0.536)		Anti-seborrheic (0.884) Anti-osteoporotic (0.848)
**207**	Antineoplastic (0.785) Prostate disorders treatment (0.758) Prostatic (benign) hyperplasia treatment (0.673) Cytoprotectant (0.656) Antineoplastic (sarcoma) (0.568) Antimetastatic (0.565) Apoptosis agonist (0.563) Proliferative diseases treatment (0.540) Antineoplastic (pancreatic cancer) (0.520) Antineoplastic (breast cancer) (0.518)	Anti-hypercholesterolemic (0.813) Hypolipemic (0.648) Cholesterol synthesis inhibitor (0.578)	Anesthetic general (0.901) Choleretic (0.725)
**208**	Antineoplastic (0.832) Prostate disorders treatment (0.740) Apoptosis agonist (0.711) Cytoprotectant (0.697) Chemopreventive (0.677) Proliferative diseases treatment (0.651) Prostate cancer treatment (0.613) Antineoplastic (breast cancer) (0.608) Antineoplastic (renal cancer) (0.552) Antineoplastic (pancreatic cancer) (0.525)	Anti-hypercholesterolemic (0.886) Lipid metabolism regulator (0.837) Hypolipemic (0.709) Cholesterol synthesis inhibitor (0.605) Atherosclerosis treatment (0.523)	Respiratory analeptic (0.969) Neuroprotector (0.924) Psychostimulant (0.707)
**209**	Antineoplastic (0.839) Chemopreventive (0.781) Apoptosis agonist (0.722) Proliferative diseases treatment (0.714) Cytoprotectant (0.654) Prostate disorders treatment (0.636) Antimetastatic (0.591)	Anti-hypercholesterolemic (0.782) Hypolipemic (0.702) Cholesterol synthesis inhibitor (0.604)	Respiratory analeptic (0.949)
**210**	Antineoplastic (0.878) Prostate disorders treatment (0.807) Prostate cancer treatment (0.721) Antineoplastic (sarcoma) (0.719) Antineoplastic (breast cancer) (0.701) Cytoprotectant (0.631) Apoptosis agonist (0.599)	Anti-hypercholesterolemic (0.538)	Cardiovascular analeptic (0.862)
**211**	Antineoplastic (0.845) Prostate disorders treatment (0.648) Antineoplastic (myeloid leukemia) (0.645) Antineoplastic (sarcoma) (0.626) Cytoprotectant (0.585) Antineoplastic (breast cancer) (0.580) Antineoplastic (renal cancer) (0.561) Antineoplastic (carcinoma) (0.521) Antineoplastic (squamous cell carcinoma) (0.521)	Hypolipemic (0.929) Lipoprotein disorders treatment (0.687)	Anti-seborrheic (0.902)
**212**	Antineoplastic (0.804) Cytoprotectant (0.719) Chemopreventive (0.678) Proliferative diseases treatment (0.622) Prostate disorders treatment (0.614) Antimetastatic (0.596)	Anti-hypercholesterolemic (0.832) Hypolipemic (0.820) Cholesterol synthesis inhibitor (0.627)	Anesthetic general (0.931) Respiratory analeptic (0.888)

* Only activities with Pa > 0.5 are shown.

**Table 15:** Biological activities of synthetic cyclobutane-containing steroids.

No.	Antitumor & Related Activity, (Pa) *	Lipid Metabolism Regulators, (Pa) *	Additional Predicted Activity, (Pa) *
**213**	Antineoplastic (0.891)		Male reproductive disfunction treatment (0.923) Aromatase inhibitor (0.717)
**214**	Antineoplastic (0.909) Prostatic (benign) hyperplasia treatment (0.663) Prostate cancer treatment (0.570)	Anti-hypercholesterolemic (0.696) Lipid metabolism regulator (0.604)	Anti-seborrheic (0.914) Respiratory analeptic (0.756)
**215**	Antineoplastic (0.860) Prostate disorders treatment (0.717) Prostatic (benign) hyperplasia treatment (0.621)		Ovulation inhibitor (0.794) Neuroprotector (0.716)
**216**	Antineoplastic (0.805) Prostatic (benign) hyperplasia treatment (0.591)	Hepatic disorders treatment (0.601) Anti-hypercholesterolemic (0.589)	Respiratory analeptic (0.871) Anti-inflammatory (0.837)
**217**	Antineoplastic (0.805) Prostatic (benign) hyperplasia treatment (0.591)	Anti-hypercholesterolemic (0.592)	Respiratory analeptic (0.874) Anti-inflammatory (0.839)
**218**	Antineoplastic (0.736) Prostate disorders treatment (0.589)	Anti-hypercholesterolemic (0.582) Atherosclerosis treatment (0.534)	Anti-seborrheic (0.915) Alopecia treatment (0.893)
**219**	Antineoplastic (0.750) Prostate disorders treatment (0.713) Prostatic (benign) hyperplasia treatment (0.501)		Anti-seborrheic (0.917) Anti-osteoporotic (0.904)
**220**	Antineoplastic (0.786) Apoptosis agonist (0.567)		Anti-seborrheic (0.924) Anti-osteoporotic (0.752)
**221**	Antineoplastic (0.854) Proliferative diseases treatment (0.588) Antimetastatic (0.552)	Hypolipemic (0.832) Anti-hypercholesterolemic (0.635) Cholesterol synthesis inhibitor (0.612)	Anti-eczematic (0.814) Anti-osteoporotic (0.657)

* Only activities with Pa > 0.5 are shown.

**Table 16:** Biological activities of steroids containing additional 5-membered ring in molecule.

No.	Antitumor & Related Activity, (Pa) *	Lipid Metabolism Regulators, (Pa) *	Additional Predicted Activity, (Pa) *
**222**	Antineoplastic (0.783) Prostate disorders treatment (0.679) Cytoprotectant (0.622) Apoptosis agonist (0.607) Antineoplastic (sarcoma) (0.603) Prostatic (benign) hyperplasia treatment (0.519) Antimetastatic (0.514) Antineoplastic (pancreatic cancer) (0.509)	Hypolipemic (0.551)	Anti-inflammatory (0.778)
**223**	Antineoplastic (0.813) Apoptosis agonist (0.683) Prostate disorders treatment (0.654) Antineoplastic (sarcoma) (0.593) Antineoplastic (pancreatic cancer) (0.541)		Anti-inflammatory (0.775) Antiprotozoal (Plasmodium) (0.622)
**224**	Antineoplastic (0.787) Prostate disorders treatment (0.685) Apoptosis agonist (0.629) Antineoplastic (sarcoma) (0.589) Prostatic (benign) hyperplasia treatment (0.550) Antineoplastic (pancreatic cancer) (0.506)		Anti-inflammatory (0.829) Antiprotozoal (Plasmodium) (0.625)
**225**	Antineoplastic (0.931) Apoptosis agonist (0.899) Antineoplastic enhancer (0.537) Cytostatic (0.519) Antineoplastic (genitourinary cancer) (0.512)		Cardiotonic (0.763) Immunosuppressant (0.683)
**226**	Apoptosis agonist (0.876) Antineoplastic (0.873) Antineoplastic (genitourinary cancer) (0.530)		Inflammatory Bowel disease treatment (0.704) Immunosuppressant (0.681)
**227**	Antineoplastic (0.885) Apoptosis agonist (0.824) Antineoplastic (genitourinary cancer) (0.550) Antimetastatic (0.513)		Cardiotonic (0.698)
**228**	Antineoplastic (0.878) Apoptosis agonist (0.861) Chemopreventive (0.717) Proliferative diseases treatment (0.581)	Anti-hypercholesterolemic (0.808) Hypolipemic (0.788) Atherosclerosis treatment (0.534)	Immunosuppressant (0.813)
**229**	Antineoplastic (0.668)		Respiratory analeptic (0.874)
**230**	Antineoplastic (0.735) Apoptosis agonist (0.545)		Anti-inflammatory (0.604)
**231**	Antineoplastic (0.846) Cytostatic (0.771) Apoptosis agonist (0.613) Antineoplastic (sarcoma) (0.526)		Hepatic disorders treatment (0.977) Macular degeneration treatment (0.882)
**232**	Antineoplastic (0.788) Apoptosis agonist (0.645)		Hepatic disorders treatment (0.937) Antiprotozoal (Plasmodium) (0.820)
**233**	Antineoplastic (0.709) Apoptosis agonist (0.632)		Anti-eczematic (0.636)
**234**	Antineoplastic (0.840) Apoptosis agonist (0.749)		Cardiotonic (0.572)
**235**	Antineoplastic (0.840) Apoptosis agonist (0.749)		Anti-inflammatory (0.637)
**236**	Apoptosis agonist (0.814) Antineoplastic (0.647) Cytoprotectant (0.613) Chemopreventive (0.564) Prostate disorders treatment (0.564)	Anti-hypercholesterolemic (0.578) Hypolipemic (0.546) Cholesterol synthesis inhibitor (0.534)	Anti-inflammatory (0.716)

* Only activities with Pa > 0.5 are shown.

**Table 17:** Biological activities of Bioactive cyclopentane- and cyclohexane-containing steroids and triterpenoids.

No.	Antitumor & Related Activity, (Pa) *	Lipid Metabolism Regulators, (Pa) *	Additional Predicted Activity, (Pa) *
**237**	Antineoplastic (0.761) Prostate disorders treatment (0.755) Prostatic (benign) hyperplasia treatment (0.683)	Anti-hypercholesterolemic (0.829) Hypolipemic (0.756) Atherosclerosis treatment (0.632)	Anesthetic general (0.901) Respiratory analeptic (0.884)
**238**	Antineoplastic (0.830) Prostatic (benign) hyperplasia treatment (0.532)		Antiprotozoal (0.781) Cardiotonic (0.773)
**239**	Antineoplastic (0.820) Prostate disorders treatment (0.784) Prostatic (benign) hyperplasia treatment (0.684) Prostate cancer treatment (0.627)		Cardiovascular analeptic (0.913)
**240**	Antineoplastic (0.910) Apoptosis agonist (0.765) Cytoprotectant (0.593) Prostate cancer treatment (0.538)		Cardiovascular analeptic (0.888)
**241**	Antineoplastic (0.765) Prostatic (benign) hyperplasia treatment (0.653) Cytoprotectant (0.641)	Anti-hypercholesterolemic (0.824) Hypolipemic (0.686) Atherosclerosis treatment (0.629)	Anti-eczematic (0.862) Anti-osteoporotic (0.826) Antiparkinsonian, rigidity relieving (0.625)
**242**	Antineoplastic (0.803) Prostatic (benign) hyperplasia treatment (0.617) Prostate cancer treatment (0.518)	Neurodegenerative diseases treatment (0.642)	Anti-osteoporotic (0.972) Anti-psoriatic (0.884)
**243**	Antineoplastic (0.797) Prostate disorders treatment (0.680) Prostatic (benign) hyperplasia treatment (0.551)	Alzheimer’s disease treatment (0.750)	Anti-osteoporotic (0.965) Anti-seborrheic (0.891) Anti-psoriatic (0.864)
**244**	Antineoplastic (0.775) Prostate disorders treatment (0.706) Cytoprotectant (0.638) Prostatic (benign) hyperplasia treatment (0.624) Apoptosis agonist (0.620)	Anti-hypercholesterolemic (0.772) Hypolipemic (0.617)	Anti-eczematic (0.846) Anti-osteoporotic (0.787)
**245**	Antineoplastic (0.777) Cytoprotectant (0.689) Prostate disorders treatment (0.677) Prostatic (benign) hyperplasia treatment (0.581)	Anti-hypercholesterolemic (0.866) Hypolipemic (0.705) Cholesterol synthesis inhibitor (0.588)	Anti-eczematic (0.840) Anti-osteoporotic (0.792)
**246**	Antineoplastic (0.918) Aromatase inhibitor (0.903) Apoptosis agonist (0.894) Prostate disorders treatment (0.699) Prostatic (benign) hyperplasia treatment (0.597) Cytoprotectant (0.597)	Anti-hypercholesterolemic (0.674) Hypolipemic (0.622)	Anti-eczematic (0.907)
**247**	Antineoplastic (0.943) Prostate disorders treatment (0.705) Prostatic (benign) hyperplasia treatment (0.601) Apoptosis agonist (0.596)		Neuroprotector (0.734) Immunosuppressant (0.650)
**248**	Antineoplastic (0.937) Aromatase inhibitor (0.903) Prostate disorders treatment (0.697) Prostatic (benign) hyperplasia treatment (0.591)		Neuroprotector (0.735) Immunosuppressant (0.654)
**249**	Antineoplastic (0.902) Prostate disorders treatment (0.740) Prostatic (benign) hyperplasia treatment (0.662) Prostate cancer treatment (0.569)		Cardiovascular analeptic (0.854) Anesthetic (0.698) Cardiotonic (0.605)
**250**	Antineoplastic (0.892) Apoptosis agonist (0.710) Prostate disorders treatment (0.662) Prostatic (benign) hyperplasia treatment (0.541)		Anti-osteoporotic (0.972)
**251**	Antineoplastic (0.742) Prostate disorders treatment (0.726) Prostatic (benign) hyperplasia treatment (0.662)	Anti-hypercholesterolemic (0.622)	Neuroprotector (0.734) Immunosuppressant (0.705)
**252**	Antineoplastic (0.769) Prostate disorders treatment (0.753) Prostatic (benign) hyperplasia treatment (0.663)		Anticonvulsant (0.733) Neuroprotector (0.727)
**253**	Antineoplastic (0.810) Prostate disorders treatment (0.726) Prostatic (benign) hyperplasia treatment (0.647)	Anti-hypercholesterolemic (0.705)	Immunosuppressant (0.764) Neuroprotector (0.749)
**254**	Antineoplastic (0.754)		Antiprotozoal (Plasmodium) (0.648)
**255**	Antineoplastic (0.774) Cytoprotectant (0.633) Prostate disorders treatment (0.572)	Hypolipemic (0.766) Anti-hypercholesterolemic (0.652) Cholesterol synthesis inhibitor (0.615)	
**256**	Antineoplastic (0.858) Proliferative diseases treatment (0.604) Apoptosis agonist (0.583) Cytoprotectant (0.561) Antimetastatic (0.549) Prostate disorders treatment (0.535)	Hypolipemic (0.838) Anti-hypercholesterolemic (0.611) Cholesterol synthesis inhibitor (0.601)	
**257**	Antineoplastic (0.694) Prostate disorders treatment (0.621) Antineoplastic (breast cancer) (0.572)		Anti-seborrheic (0.928) Cardiovascular analeptic (0.674)
**258**	Antineoplastic (0.854) Prostatic (benign) hyperplasia treatment (0.621)	Anti-hypercholesterolemic (0.682)	Neuroprotector (0.756) Acute neurologic disorders treatment (0.741)
**259**	Antineoplastic (0.845) Apoptosis agonist (0.654) Prostatic (benign) hyperplasia treatment (0.585)	Hypolipemic (0.548)	Cardiotonic (0.917) Antiarrhythmic (0.809)
**260**	Antineoplastic (0.823) Prostate disorders treatment (0.746) Prostatic (benign) hyperplasia treatment (0.615)		Anesthetic general (0.841)
**261**	Antineoplastic (0.715) Prostate disorders treatment (0.701) Prostatic (benign) hyperplasia treatment (0.619)		Anesthetic general (0.712)
**262**	Antineoplastic (0.834)	Anti-hypercholesterolemic (0.794)	Anesthetic general (0.805)
**263**	Antineoplastic (0.796) Apoptosis agonist (0.723) Prostate disorders treatment (0.676)	Anti-hypercholesterolemic (0.527)	Anti-osteoporotic (0.934) Anti-seborrheic (0.918)
**264**	Antineoplastic (0.757) Prostate disorders treatment (0.658) Apoptosis agonist (0.550) Prostatic (benign) hyperplasia treatment (0.503)		Spasmolytic, urinary (0.961)

* Only activities with Pa > 0.5 are shown.

**Table 18:** Bioactive synthetic steroids containing an additional 5- or 6-membered ring in molecule.

No.	Antitumor & Related Activity, (Pa) *	Lipid Metabolism Regulators, (Pa) *	Additional Predicted Activity, (Pa) *
**265**	Antineoplastic (0.933) Apoptosis agonist (0.667)		Antimitotic (0.843)
**266**	Antineoplastic (0.942) Apoptosis agonist (0.619) Antineoplastic (sarcoma) (0.510)		Antimitotic (0.848)
**267**	Antineoplastic (0.934) Apoptosis agonist (0.890) Cytostatic (0.688) Antineoplastic (sarcoma) (0.647) T cell inhibitor (0.608) Prostate disorders treatment (0.606) Antineoplastic (pancreatic cancer) (0.573)		Antimitotic (0.829) Antiprotozoal (Plasmodium) (0.650)
**268**	Antineoplastic (0.936) Apoptosis agonist (0.720) Antimetastatic (0.515) Antineoplastic (pancreatic cancer) (0.504)		Antimitotic (0.849)
**269**	Antineoplastic (0.922) Apoptosis agonist (0.641) Antimetastatic (0.515)		Antimitotic (0.819) Antiprotozoal (Plasmodium) (0.694)
**270**	Antineoplastic (0.929) Apoptosis agonist (0.669) Antineoplastic (renal cancer) (0.570)		Antimitotic (0.853)
**271**	Antineoplastic (0.930) Apoptosis agonist (0.753) Cytostatic (0.735) Antineoplastic (renal cancer) (0.603) Antineoplastic (sarcoma) (0.602) Antineoplastic (pancreatic cancer) (0.551) Antineoplastic (lymphocytic leukemia) (0.548) Antineoplastic (myeloid leukemia) (0.529) Antineoplastic (genitourinary cancer) (0.523)		Antimitotic (0.776) Immunosuppressant (0.665)
**272**	Antineoplastic (0.933) Apoptosis agonist (0.805) Antimetastatic (0.535) Antineoplastic (pancreatic cancer) (0.524)		Antimitotic (0.808) Immunosuppressant (0.745)
**273**	Antineoplastic (0.934) Apoptosis agonist (0.805) Antineoplastic (sarcoma) (0.530) Antineoplastic (pancreatic cancer) (0.524)		Antimitotic (0.804) Immunosuppressant (0.731) Antiprotozoal (Plasmodium) (0.668)
**274**	Antineoplastic (0.875) Apoptosis agonist (0.728) Chemopreventive (0.693) Prostate disorders treatment (0.670) Proliferative diseases treatment (0.659) Anticarcinogenic (0.630) Antineoplastic (breast cancer) (0.551) Antineoplastic (pancreatic cancer) (0.540) Prostatic (benign) hyperplasia treatment (0.526) Antineoplastic (sarcoma) (0.517)	Anti-hypercholesterolemic (0.858) Hypolipemic (0.767) Cholesterol synthesis inhibitor (0.608) Atherosclerosis treatment (0.600) Lipid metabolism regulator (0.590)	Anti-ischemic, cerebral (0.932) Antiprotozoal (Leishmania) (0.559)
**275**	Chemopreventive (0.966) Apoptosis agonist (0.896) Antineoplastic (0.866)	Hypolipemic (0.575)	
**276**	Chemopreventive (0.958) Apoptosis agonist (0.842) T cell inhibitor (0.620)	Hypolipemic (0.540)	

* Only activities with Pa > 0.5 are shown.
